# Supplementary material for: Study protocol on risk factors for the diagnosis of gestational diabetes mellitus in different trimesters and their relation to maternal and neonatal outcomes (GDM-RIDMAN)
Source: BMJ Open. 2022 Jul 26;12(7):e052554. doi: 10.1136/bmjopen-2021-052554 (PMC9330332; doi:10.1136/bmjopen-2021-052554)
Supplement: Author's manuscript [file bmjopen-2021-052554.draft_revisions.pdf]

BMJ Open

BMJ Open is committed to open peer review. As part of this commitment we make the peer review history of every article we publish publicly available.

When an article is published we post the peer reviewers' comments and the authors' responses online. We also post the versions of the paper that were used during peer review. These are the versions that the peer review comments apply to.

The versions of the paper that follow are the versions that were submitted during the peer review process. They are not the versions of record or the final published versions. They should not be cited or distributed as the published version of this manuscript.

BMJ Open is an open access journal and the full, final, typeset and author-corrected version of record of the manuscript is available on our site with no access controls, subscription charges or pay-per-view fees (http://bmjopen.bmj.com).

If you have any questions on BMJ Open's open peer review process please email info.bmjopen@bmj.com

BMJ Open

Risk factors for the diagnosis of gestational diabetes mellitus in different trimesters and their relation to maternal and neonatal outcomes (GDM-RIDMAN): a retrospective cohort and nested case-control study

Journal:	*BMJ Open*
Manuscript ID	bmjopen-2021-052554
Article Type:	Protocol
Date Submitted by the Author:	19-Apr-2021
Complete List of Authors:	Yap, Pamela; Universiti Putra Malaysia Fakulti Perubatan dan Sains Kesihatan,<br>Papachristou Nadal, Iliatha; London School of Hygiene and Tropical Medicine, Faculty of Epidemiology and Population Health; King's College London<br>Rysinova, Veronika; King's College London Institute of Psychiatry Psychology and Neuroscience<br>Basri, Nurul Iftida; Universiti Putra Malaysia Fakulti Perubatan dan Sains Kesihatan, Department of Obstetrics and Gynaecology<br>Samsudin, Intan; Universiti Putra Malaysia Fakulti Perubatan dan Sains Kesihatan, Department of Pathology<br>Forbes, Angus; King's College London, Division of Care in Long-term Conditions<br>Noor, Nurain; Hospital Putrajaya Malaysia<br>Supian, Ziti; Klinik Kesihatan Seri Kembangan<br>Hassan, Haslinda ; Klinik Kesihatan Puchong<br>Paimin, Fuziah; Klinik Kesihatan Putrajaya Presint 9<br>Zakaria, Rozita; Klinik Kesihatan Putrajaya Presint 18<br>Mohamed Alias, Siti; Klinik Kesihatan Kajang<br>Ismail Bukhary, Norizzati; Klinik Kesihatan Bangi<br>Benton, Madeleine; King's College London, Department of Psychological Medicine<br>Ismail, Khalida; Institute of Psychiatry, Kings College London<br>Chew, Boon How; Universiti Putra Malaysia, Family Medicine; Hospital Pengajar UPM (UPM Teaching Hospital), Clinical Research Unit
Keywords:	Diabetes in pregnancy < DIABETES & ENDOCRINOLOGY, Clinical chemistry < PATHOLOGY, Depression & mood disorders < PSYCHIATRY

SCHOLARONE™
Manuscripts

Title: Risk factors for the diagnosis of gestational diabetes mellitus in different trimesters and their relation to maternal and neonatal outcomes (GDM-RIDMAN): a retrospective cohort and nested case-control study

Authors:

Pamela Phui Har Yap[1], Iliatha Papachristou[4], Veronika Rysinova[7], Nurul Iftida Basri[2], Intan Nureslyna Samsudin[3], Angus Forbes[5], Nurain Mohd Noor[8], Ziti Akthar Supian[9], Haslinda Hassan[10], Fuziah Paimin[11], Rozita Zakaria[12], Siti Rohani Mohamed Alias[13], Norizzati Bukhary Ismail Bukhary[14], Madeleine Benton[6], Khalida Ismail[6], Boon How Chew[1,15].

Affiliations:

[1]Department of Family Medicine, Faculty of Medicine and Health Sciences, Universiti Putra Malaysia, 43400 Serdang, Selangor, Malaysia.

[2]Department of Obstetrics and Gynaecology, Faculty of Medicine and Health Sciences, Universiti Putra Malaysia, 43400 Serdang, Selangor, Malaysia.

[3]Department of Pathology, Faculty of Medicine and Health Sciences, Universiti Putra Malaysia, 43400 Serdang, Selangor, Malaysia.

[4]King's College London / London School of Hygiene and Tropical Medicine, UK.

[5]Primary and Intermediate Care Section, King's College London.

[6]Department of Psychological Medicine, King's College London.

[7]Institute of Psychiatry, Psychology & Neuroscience, Kings College London.

[8]Hospital Putrajaya, Jalan P9, Presint 7, 62250 Putrajaya, Wilayah Persekutuan Putrajaya, Malaysia.

[9] Klinik Kesihatan Seri Kembangan, Jalan Besar, Taman Muhibbah, 43300 Seri Kembangan, Selangor, Malaysia.

[10]Klinik Kesihatan Puchong Batu 14, 1, Jalan PU 5, Taman Puchong Utama, 47100 Puchong, Selangor, Malaysia.

[11]Klinik Kesihatan Putrajaya Presint 9, 1, Jalan P9e, Presint 9, 62250 Putrajaya, Wilayah Persekutuan Putrajaya, Malaysia.

[12]Klinik Kesihatan Putrajaya Presint 18, Jalan P18c 1, Presint 18, 62150 Putrajaya, Wilayah Persekutuan Putrajaya, Malaysia.

[13]Klinik Kesihatan Kajang, Jalan Semenyih, Bandar Teknologi Kajang, 43500 Kajang, Selangor, Malaysia.

[14]Klinik Kesihatan Bangi, Jalan 3/1, Seksyen 3 Bandar Baru Bangi, 43650 Bandar Baru Bangi, Selangor, Malaysia.

For peer review only - http://bmjopen.bmj.com/site/about/guidelines.xhtml

For peer review only

[15]Clinical Research Unit, Hospital Pengajar Universiti Putra Malaysia (HPUPM Teaching Hospital), Persiaran MARDI - UPM, 43400 Serdang, Malaysia

Corresponding author:

Boon-How Chew, Department of Family Medicine, Faculty of Medicine and Health Sciences, Universiti Putra Malaysia, 43400 Serdang, Selangor, Malaysia. Email: chewboonhow@upm.edu.my.

Word count: 5716 words from Introduction to Dissemination plan (excluding tables)
Word count: 5218 words from Introduction to Discussion (excluding Ethics & Dissemination, and tables)

For peer review only

ABSTRACT (300 words)

Introduction: Gestational diabetes mellitus (GDM) is often associated with adverse pregnancy outcomes. All pregnant women are usually risk stratified for GDM screening according to a clinical practice guideline (CPG) on their first antenatal clinic visit. However, the association of the risk factors with GDM diagnosis, maternal and neonatal health outcomes is less established compared to women without GDM. We aims to examine the diagnostic accuracy of the latest Malaysian CPG-based risk factors and to examine novel risk factors for a GDM diagnosis in the first and second trimester, in relation to glucose regulation and impact on maternal and neonatal health outcomes.

Methods and analysis: This retrospective cohort and nested case-control study at six public health clinics in Selangor and Putrajaya is based on medical records, and questionnaire survey of women between 2-12 month postpartum. Estimated required sample size is 876 complete records (292 cases, 584 control, at a ratio of 1:2). An oral glucose tolerance test will be used to identify glucose dysregulation, and maternal and neonatal outcomes will include maternal weight gain, pre-eclampsia, polyhydramnios, mode of delivery, per-term birth, postpartum haemorrhage and perineal tear, and mode of birth, birth weight, gestational age at birth, Apgar score, congenital anomaly, congenital hypothyroidism, neonatal death or stillbirth, hypoglycaemia and hyperbilirubinemia. Psychosocial measures include the World Health Organization Quality of Life: Brief Version Questionnaire, mother-infant bonding (14-item Postpartum Bonding Questionnaire and 19-item Maternal Postnatal Attachment Scale), anxiety (7-item Generalized Anxiety Disorder), depression (9-item Patient Health Questionnaire), and stress (Perceived Stress Scale symptoms). Sociodemographic data and clinical variables will be analysed descriptively. Comparative incidences of maternal and neonatal health outcomes and the comparative prevalence of the psychosocial outcomes between women with GDM and without GDM will be reported. The current and novel GDM risk factors and outcomes will be modelled using multivariable regression analysis.

Keywords: gestational diabetes mellitus, risk factors, diagnosis, maternal and neonatal outcomes, antenatal care, postpartum, Malaysia

For peer review only - http://bmjopen.bmj.com/site/about/guidelines.xhtml

Article summary:

Strengths and limitations of this study

- This study provides an opportunity to confirm and explore the conventional CPG-based and potential new risk factors to better predict the diagnosis of GDM, either at the first or/and second trimester, and on maternal and neonatal outcomes.
- The effect of multiple pregnancies among other novel risk factors on the diagnosis of GDM which were often excluded from earlier studies will be examined.
- Results will suggest the best modelled risk profiles that predict the diagnosis of GDM that may lead to improved identification of at-risk women and allow earlier treatment initiation.
- Participants are mainly from the urban areas of Selangor and Putrajaya, and may not be representative of a larger population.
- Recruitment of fewer participants than expected could be due to the movement restriction and clinic operation caused by Covid-19 pandemic, reduced number of pregnancies and postpartum women, and limited by the study duration.

For peer review only - http://bmjopen.bmj.com/site/about/guidelines.xhtml

INTRODUCTION

Gestational diabetes mellitus (GDM) is a form of hyperglycaemia, where pregnant women experience glucose intolerance for the first time during the pregnancy[1–3]. Efficient GDM screening and accurate diagnosis allows early management and treatment could reduce adverse pregnancy outcomes for both mother and child[4–6].

Asian women is at a higher risk for GDM compared to Caucasian women[7,8]. A systematic review and meta-analysis reported that the prevalence of GDM in Malaysia is in the top five Eastern and Southeast Asian countries with approximately one in nine pregnant women had GDM (11.8%)[8]. The latest National Obstetric Registry 2016-2017 reported the prevalence of GDM with adverse outcomes in Malaysia range from 10.8% to 19.3%[9]. Spontaneous miscarriage and caesarean section are the most frequently reported adverse outcomes in GDM women compared to healthy women, 5.9% vs. 2.6% and 28.5% vs. 18.8%, respectively[10]. Other adverse maternal outcomes include birth trauma, postpartum haemorrhage, pre-eclampsia, and hypertension (≥140/90 mmHg) after 20th week of gestation with proteinuria[10,11]. Common neonatal adverse outcomes include foetal macrosomia, hypoglycaemia, prematurity, shoulder dystocia, hyperbilirubinemia and admission to intensive care units[11,12]. Prevalence of macrosomic babies and neonatal hypoglycaemia among GDM mother in Malaysia in 2018 are 4.8%, and 1.7%, respectively[12]. Post-diagnosis postprandial glucose levels are associated with macrosomia and large for gestational age (LGA), premature delivery, gestational hypertension and hyperbilirubinemia[13–15].

At both the local and international level screening methods and diagnosis of GDM remains debatable. High fasting plasma glucose (FPG) level significantly increases the risk for LGA foetus, primary caesarean section and development of GDM in later pregnancy[16]. Several studies from Israel and Asian population supports FPG ≥6.10 mmol/L at first trimester as the predictor tools for GDM development in later pregnancy[17,18]. Another study in China suggested FPG 6.1-6.9 mmol/L were more accurate and reliable at early pregnancy (before 24th week) for GDM diagnosis. This differs from the International Association of Diabetes and Pregnancy Study Group (IADPSG) cut-off values of FPG ≥5.1 mmol/L[18,19]. However, this is less well accepted because many perceive FPG ≥5.1 mmol/L is a false alarm, as pregnancy advance in week, FPG level decreases[18]. Glycosylated hemoglobin A1c (HbA1c) is not widely used in practice as the value can be unreliable due to many confounding conditions such as hemoglobinopathy[20] although studies have shown the predictive value of HbA1c ≥6.0% of GDM development[5] and adverse neonatal outcomes[10,21,22].

Malaysia practices selective risk-based screening and one-step diagnosis for GDM for early diagnosis and management. All pregnant women will be risk stratified according to the Malaysia 2017 Clinical Practice Guidelines (CPG) on Management of Diabetes in Pregnancy[23]. Women who are perceived to be at risk of GDM will undergo 75g oral glucose tolerance test (OGTT) as immediately

For peer review only - http://bmjopen.bmj.com/site/about/guidelines.xhtml

as in the next appointment between 24th to 28th week of gestation[23]. The test requires women to stay fasted from food and drink for at least 8 – 12 hours for FPG and 2 hours after the oral glucose intake. The one-step diagnosis approach of GDM will diagnose GDM when at least any one single abnormal reading is observed at fasting or 2-hour from 75g OGTT[19,23,24]. There are some challenges in full OGTT compliance which include long hours of fasting, drinking of glucose water, vomiting and defaulting the 2-hour post-prandial (2-HPP) plasma glucose test[25–27]. However, there is uncertainty in terms of the state of OGTT compliance and completion rate at the first or second trimester. Similarly, there is a lack of information about the completion rate of OGTT and its association with adverse pregnancy outcomes among pregnant women before the GDM diagnosis[11]. Therefore, adherence of OGTT is equally important during and after pregnancy for GDM women.

The common risk factors of GDM include age ≥25 years old, body mass index >27 kg/m^2, previous history of GDM, first degree relative with diabetes mellitus, history of macrosomia (birth weight >4 kg), bad obstetric history (unexplained intrauterine death, neural tube defects, cardiac defects and shoulder dystocia), glycosuria ≥2+ on two occasions, current obstetric problems (essential hypertension, pregnancy-induced hypertension (≥140/90 mmHg), polyhydramnios and current use of corticosteroids[23] (see Table 1). Other potential risk factors for GDM include polycystic ovarian syndrome (PCOS) (OR 2.33, 95% Cl 1.72-3.17), multiple pregnancies (OR 1.37%, 95% Cl 1.24-1.52), pre-term birth (OR 1.93, 95% Cl 1.21-3.07)[24], maternal gestational weight gain (aOR 3.38%, 95% Cl 1.83-6.24)[28], and maternal smoking status (OR 1.22, 95% Cl 1.08 – 1.38)[29]. The prevalence of PCOS among Malaysian working women age 18 – 49 years was 12.6 %[30] but PCOS is not consider as a risk factor in the Malaysian CPG. Multiple pregnancies is associated with higher risk for GDM due to a higher weight gain rather than the number of foetus[31]. The rate of multiple pregnancies deliveries among GDM women in Malaysia range approximately from 12% to 30%[32,33]. Many GDM studies have excluded multiple pregnancies in their eligibility criteria, so the risk of multiple pregnancies on GDM is not clear. There are conflicting evidence on smoking and GDM[34,35].

Psychosocial aspects in the postpartum period

There is increasing evidence demonstrating that psychosocial factors, such as depressive symptoms, anxiety, high perceived stress, poor quality of life (QoL) and weakened mother-child bonding play an important role in GDM and adverse neonatal outcomes[12,36–38]. A systematic review demonstrated that GDM respondents consistently showed significantly lower quality of life both short- and long-term compared to healthy pregnant participants[37] and whereas the QoL scores significantly improved after pregnancy in healthy women, general health perception in GDM women remained significantly lower[39,40]. The estimated prevalence of depression, anxiety, stress symptoms and poor QoL in GDM women in Malaysia ranges from 10.2% to 39.9%[12].

For peer review only - http://bmjopen.bmj.com/site/about/guidelines.xhtml

Additionally, the odds of GDM were found to be 13 folds higher among women experiencing high rates of perceived stress during pregnancy compared to those with low perceived stress among Indian women[41], suggesting that high perceived stress is a potential risk factor for GDM development. Similarly, a recent systematic review and meta-analysis investigating 62 studies indicated that there was an increased risk of depression and anxiety symptoms around the time of GDM diagnosis and in the postnatal period[42]. Weakened mother-child bonding was previously shown to have a statistically significant association with mother's depression and anxiety symptoms and these variables also affected mother-child bonding[36]. Mother-child bonding is crucial to the mental growth and development of infants[43]. Given the potential link between GDM and psychosocial factors, investigation and integration of physical and mental health factors in empirical studies and interventions with women experiencing GDM in Malaysia is therefore vitally important and could improve short- and long-term outcomes for women and their children[42].

Conceptual framework

The independent variables are risk factors of GDM including both the CPG-based (Table 1) and the potential risk factors (Table 2) based on the literature. The first dependent variables is the diagnosis of GDM in the first or second trimester as recorded on the maternal records. The second separate dependent variables in this study is the maternal and neonatal health outcomes including the maternal psychological wellbeing. The effect of glycaemic patterns after GDM diagnosis on the maternal and neonatal health outcomes is believed to be present and will be examined. Additionally, we aim to describe the incidences of the maternal and neonatal outcomes during pregnancy and in the postpartum period in women with GDM and without GDM. Figure 1 show the overall conceptual framework of this study.

Since there are uncertainties about the differences in risk factor profiles and the antenatal hyperglycaemia patterns, and also the difference on pregnancy outcomes of those who completed the OGTT in the first or second trimester with a GDM diagnosis, this study aims to examine and quantify the effects of the current recommended CPG-based risk factors for GDM diagnosis and to compare the risk factor profiles between those associated with GDM diagnoses at the first and second trimester. We will also examine the effects of other potential new risk factors on the actual diagnosis of GDM. Additionally, we will investigate the impacts of all the risk factors and the post-diagnosis blood sugar profiles on the maternal and neonatal outcomes.

For peer review only - http://bmjopen.bmj.com/site/about/guidelines.xhtml

METHOD AND ANALYSIS

This retrospective cohort and nested case-control study will be based on antenatal and postnatal medical records, and a questionnaire survey of postpartum women who delivered within the past 2-12 months. The five study objectives are:

1. To compare the risk factor profiles of women who undergone OGTT and diagnosed with GDM in the first and second trimesters.
2. To describe the occurrences of the maternal health outcomes during pregnancy and in the postpartum period between those with GDM and without GDM.
3. To describe the incidence of the neonatal health outcomes of mothers with GDM and without GDM.
4. To identify and quantify risk factors (including blood sugar profiles) that are associated with any maternal complications.
5. To identify and quantify risk factors (including blood sugar profiles) that are associated with any neonatal complications.

Settings

Data will be collected from six participating public health clinics in Selangor and Putrajaya over an approximately 5 months. These health clinics are attended by pregnant women of different ethnicities, provide maternal and childcare services with in-house laboratory services, and availability of GDM registry. The standard care processes in these clinics are that women will be seen by nurses and medical officers or a family medicine specialist when needed for a further care. GDM women usually have a follow-up appointment every two weeks for blood sugar level monitoring and pregnancy progress consultation. In post-partum, the appointment ranges from four to six weeks for family planning counselling and a repeat for the OGTT[10,44].

Pre-prandial and post-prandial glucose tests are blood sugar profile tests used for GDM monitoring. A pre-prandial plasma glucose should be ≤5.3 mmol/L[23,45], and post-prandial values at 1-hour and 2-hour are ≤7.8 mmol/L and ≤6.7 mmol/L to be considered as optimal, respectively[23].

Participants

The eligible participants are Malaysian women age ≥18 years old, had undergone OGTT during the last pregnancy, both singleton and multiple pregnancies, with a baby age at least two months and above *(include pre-term birth between 23 – 37 week of gestation but chronological age at least two months)* or has any miscarriage during last pregnancy, receiving most antenatal care at the participating clinics in the last pregnancy and return for a postnatal follow-up at the participating clinics. Malaysian women with pre-existing type 1 or type 2 diabetes and overt diabetes will be excluded from this study.

Instruments

For peer review only - http://bmjopen.bmj.com/site/about/guidelines.xhtml

GDM screening and diagnosis are based on the Malaysian 2017 CPG on Management of Diabetes in Pregnancy[23]. Pregnant women with either one of the abnormal OGTT results, FPG ≥5.1 mmol/L or 2-HPP ≥7.8 mmol/L will be diagnosed as GDM[23]. All data will be retrieved from the antenatal home-based record, and if necessary from the baby's record book, clinic-based record, GDM registry book, healthcare electronic medical records and laboratory records that are available at each participating centre. If there is insufficient or missing data from the records, we may call the participants for clarification. All variables will be recorded in a structured case record form. All data retrieved will be labelled, stored safely in hard drive and locked with password. The independent and dependent variables include the following:

a. Mother's risk factors: age ≥25 years old, at booking BMI >27 kg/m^2, history of GDM, first degree family with diabetes mellitus, previous baby with birth weight >4kg, bad obstetrics history (unexplained intrauterine death, congenital abnormalities as such neural tube defects, cardiac defects and shoulder dystocia), glycosuria ≥2+ on two occasions, medical disorders (hypertension ≥140/90 mmHg, polyhydramnios and on corticosteroid medication), multiple pregnancies, smoking status, miscarriage happens before 23 week on previous or most present pregnancy, and history of PCOS.
b. OGTT result, all blood sugar profiles (blood glucose testing performed at the clinic or home-monitoring) and HbA1c level.
c. Delivery records include:
    i. maternal outcomes (gestational weight gain, pre-eclampsia, polyhydramnios, mode of delivery, gestational age at birth (pre-term birth) and complication in pregnancy includes postpartum haemorrhage and perineal tear).
    ii. neonatal outcomes (mode of birth, birth weight, gestational age at birth (LGA or SGA), Apgar score, congenital anomaly, congenital hypothyroidism from TSH and T4 levels, neonatal death and stillbirth, hypoglycaemia from plasma glucose level, and hyperbilirubinemia from serum bilirubin level).
d. Psychosocial measures include quality of life[46], mother-infant bonding[47–49], anxiety and depression symptoms[50,51], and perceived stress[52] (Table 3).

<u>Cultural adaptation and validation process for questionnaires</u>

The 14-item Postpartum Bonding Questionnaire (PBQ-14) and 19-item Maternal Postnatal Attachment Scale (MPAS-19) will be translated from English to Malay by bilingual translators. Two forward translations from English to Malay will be produced and then another bilingual translator will translate the scale back into English while being blinded to the original English version of the questionnaires[63]. The two Malay versions for the scale will be compared with the original and back-

For peer review only - http://bmjopen.bmj.com/site/about/guidelines.xhtml

translated English version. Based on discussion and consensus by an expert committee, the most appropriate Malay version will be developed and chosen in this study. The expert committee comprises three Malay women with a history of GDM, bilingual (English and Malay) two family physicians, two psychologists and one obstetrician & gynaecologist.

Principal component analysis (PCA) will be used, utilising the Orthogonal rotation (Varimax) to determine the subscales. Preliminary analysis of the PCA output will be made to investigate multicollinearity, sampling adequacy using the Kaiser-Meyer-Olkin statistic and Bartlett's test of sphericity. Internal consistency will be calculated with Cronbach's alpha values for all subscales and 2-week test-retest reliability will be examined with intra-class correlation coefficients (ICC). A Cronbach's alpha value of at least 0.75 indicates good internal consistency of the questionnaire[64]. ICC of at least 0.7 is preferred for a sample size of >50 subjects to estimate the test–retest reliability[65,66].

To check for construct validity (hypothesis-testing validity)[67], associations with GDM status, maternal outcomes (weight gain, pre-eclampsia, polyhydramnios, mode of delivery, pre-term birth, postpartum haemorrhage, perineal tear, retained placenta, postnatal OGTT result), neonatal outcomes (mode of birth, birth weight, Apgar score, congenital abnormalities, shoulder dystocia, neonatal hypothyroidism, neonatal hypoglycaemia, neonatal hyperbilirubinemia, admission to NICU) and the other psychosocial measures (WHOQOL-Bref, GAD-7, PHQ-9, and PSS-10) will be examined. Lastly, PBQ-14, and MPAS-19 will be examined against each other. We hypothesis that maternal feelings of bonding are moderately correlated ($r \geq 0.5$) and associated with maternal mood[68,69]. All statistical analyses will use Statistical Package for Social Sciences (SPSS) version 26.0 (IBM, Chicago, IL) and p value >0.05 will be considered statistically significant.

Sampling process

Study will start at all six participating health clinics at the same time. All eligible participants will be invited to participate in the study. All women with GDM will be the study case. For every case of GDM, we will sample two control of women who had undergone OGTT in same clinic at about the same period of gestation but without a diagnosis of GDM. Women who consented and signed the consent form will leave their home-based health record for the researcher for a period of 1 – 2 months. They will self-administer the questionnaires (about 30 minutes to complete) in either Malay or English according to their preference and return it before they leave the clinic. A trained research assistant will be at each participating clinic to facilitate the data collection and to clarify query on the questionnaire. Every booklet will be inserted a bookmark with a unique code and kept in safe spaces in the clinics until collected data retrieval. In return, the participating women will receive a copy of the Participant Information Sheet, signed consent formed, and the same duplicate bookmark with the same code attached to their health record. The bookmark contains the study and the researcher contact information. Participants can request for early return of their home-based record at any time during the

For peer review only - http://bmjopen.bmj.com/site/about/guidelines.xhtml

study period, and it shall be returned to them immediately through the clinic or registered mail. At the end of the study, all home-based records will be returned to each clinic and the women will be informed for collection. They will present the duplicate bookmark for verification to collect their health records.

At each participating clinic, we will extract data from the OGTT records, and only request access to the health records of the consented patients to verify necessary information including the history of miscarriage or pre-term birth. Figure 2 show the overview of the procedures in data collection.

We will also re-invite at least 50 participants each from both women with GDM and without GDM to complete the PBQ-14 and MPAS-19 for the 2-week intra-rater test-retest reliability testing[67]. Assuming 50% response rate, the first 100 women with and without GDM (total n= 200) will be invited to participate in this reliability test. The same questionnaire will be given to the selected participants in an envelope that contains a second self-addressed and stamped envelope. Completed questionnaire will be posted to the investigator. Additionally, we will send a Google form link to the questionnaires to the selected participants in the reminder message at 2-week.

Sample size estimation

Based on previous studies, the prevalence of GDM with adverse outcomes range from 5-27.9%[4,11]. We use GPower 3.1.9.7[70] with 0.90 power and significance at two-sided α of 0.05 to estimate the smallest difference in maternal or neonatal complication rate between GDM with optimal glycaemic control and suboptimal control to be at 15%, the required sample size is 312 (104 cases, 208 control). On another estimation, the required sample size to estimate 10% (as the lowest possible proportion among all the risk factors) either the history of PCOS with the reported prevalence rate 12.6%[30] or multiple pregnancies with reported incidence of 12-30% among GDM women[32,33] with the power 0.90 and α 0.05 at two tails is 263. Taking into consideration of about 10% of incomplete or missing data in the home-based records, the sample size needed is 292 (263/0.90) cases and 584 (526) control at the ratio of 1:2. Knowing the average number of pregnancies at each of the six participating clinics (200-300 cases and 1500-3000 controls per year) and the required 1:10 ratio of independent variables to numbers of dependent variable to run a multiple logistic regression with 14 independent variables on GDM with any maternal or neonatal complication rate of 40%[71], the study is deem feasible. Therefore, we will collect the home-based records from the postpartum women at the participating centres until at least 876 records have been collected.

Data analysis plan

All data analysis will use SPSS version 26.0 (IBM, Chicago, IL). Data entered will be cleaned and checked for the missing, extreme and suspicious values. These may be verified with the respondents or omitted as missing values. Once the missing data determined to be missing at random, multiple

For peer review only - http://bmjopen.bmj.com/site/about/guidelines.xhtml

imputations with ten runs may be conducted to replace the missing data. The complete case analysis will be conducted if the sample size achieves 789 at minimum.

We will use a descriptive analysis to summarize the sociodemographic data and clinical variables according to the diagnosis of GDM at first and second trimester at 24th to 28th weeks of gestation. All glycaemic biomarkers including blood sugar profiles will be reported in the trimesters according to the outcomes (normal vs. adverse). Comparisons of mean levels for continuous variables analyse using Student's t-test and Chi-square test for categorical variables. The equivalent non-parametric tests will be used for data with non-normal distribution. We will report the proportion of postnatal women who had completed OGTT in the first trimester, those who completed OGTT in the second trimester and among each of this group the proportions offered OGTT twice or more (when they need to repeat the test).

To achieve the first objective, we will calculate the proportion of risk factors that are identified according to the trimester when GDM diagnosis is made. We will compare the risk factors profile of women completed OGTT and diagnosed GDM at first trimester to those undergone the OGTT but without GDM. A similar analysis will also be conducted to compare the risk factor profiles of women with and without GDM who were only offered OGTT during the second trimester and not in the first trimester. We will report the specificity, sensitivity, positive and negative predictive values of the CPG-based risk factors and to examine the novel risk factors, separately and combined, for a GDM diagnosis in the first and second trimester, respectively. Additionally, we will compare the risk factors profile of women diagnosed with GDM at the first and second trimester. We will model the risk factor profiles that best predict the GDM diagnosis at the first and second trimester, respectively, using multiple logistic regression.

We will report the comparative incidences of the maternal health outcomes during pregnancy and labour, and the comparative prevalence of the psychosocial outcomes in the postpartum period between the women with GDM and without GDM (second objective). Similarly, we will report the comparative incidences of the neonatal outcomes between the women with GDM and without GDM (third objective).

The fourth objective can be achieved by comparing the adjusted R^2 values of multivariable logistic regression models consisting of the CPG-based risk factors and models with the added new potential risk factors for GDM diagnosis, including the documented glycaemic biomarkers on the outcome of maternal complications as a whole or by each complication. If sample size allows, this analysis will be conducted separately for GDM diagnosed at first and second trimester to examine its effect on the maternal complications.

Analysis step to measure the fifth objective is similar as to the fourth objective by comparing the adjusted R^2 values of multivariable logistic regression models consisting of the CPG-based risk

For peer review only - http://bmjopen.bmj.com/site/about/guidelines.xhtml

factors and models with the added new potential risk factors for GDM diagnosis, including documented glycaemic biomarkers on neonatal health outcomes.

The current risk factors for GDM will be assessed univariably and multivariably of their effect on the diagnosis of GDM at first and second trimester (24th to 28th weeks) using the R^2 and adjusted R^2. The factors from the demographic and clinical variables on each of the GDM diagnosis will be estimated in univariable logistic regression analyses. Any of this factor with a *P*-value <0.20 from will be included in the multiple logistics regression analysis[72]. The analyses may be conducted by blocks of risk factors such as sociodemography (age, BMI and smoking), family history and past medical history, current antenatal medical problems (glycosuria, obstetric medical conditions and weight gain) if sample size is less than desirable. Multicollinearity between any independent variables will be checked according to the tolerance <0.4 (Variance inflation factors ≥2.5). In the presence of multicollinearity, the more critical or essential variable from clinical perspectives will be selected for use in the final regression analysis. All models, Q-Q plots for normality, the residual plots for linearity and homogeneity assumptions and model fitting will be checked. Same statistical strategy may be conducted to model the independent predictors on the maternal and neonatal outcomes. The maternal outcomes will be combined as a whole or separately if sample size allows, and the maternal psychosocial wellbeing measures will be analysed separately. Similarly, if sample size allows, we will analyse for the neonatal outcomes.

Patient and Public Involvement

This is one of non-experimental studies in the MYGODDESS Project (https://rb.gy/ccztw5) where women with GDM during pregnancy and in postpartum periods were interviewed on important barriers and facilitators of self-care. Women with a history of GDM are involved in the face and content validity testing of the PBQ-14 and MPAS-19 questionnaires. Their opinions on the study design, conduct, reporting and dissemination of results will be sought at appropriate time during the study.

DISCUSSION

This retrospective cohort study incorporates a nested case-control design, providing an opportunity to confirm and explore the conventional CPG-based and potential risk factors to better predict the diagnosis of GDM, either at the first or/and second trimester. This is one of the core outcomes to be included in GDM prevention and treatment research[73]. Additionally, we will also examine multiple pregnancies on diagnosis of GDM, which was often excluded from earlier similar studies. The risk profiles that best predict the diagnosis of GDM will be modelled and examined of their impacts on the maternal and neonatal health outcomes. This evidence is imperative to improve the identification of at-risk women and earlier treatment for GDM. This study would help healthcare practitioners and women with GDM to better understand the effect of both the currently 'recommended' and potentially

For peer review only - http://bmjopen.bmj.com/site/about/guidelines.xhtml

'new' important risk factors of GDM, patterns of glycaemic control and association with health outcomes in both the women and neonates. Primarily, we may verify the diagnostic accuracy or to quantify the effect of the current CPG-based risk factors and potential risk factors on GDM diagnosis and on both maternal and neonatal health outcomes. This is potentially impactful on the decision rule of the existing practice. If a delay in the completion of OGTT is one of the possible causes of a delayed GDM diagnosis and treatment, the study would determine the level of OGTT completion, and identify the risk profiles of women and their association with any maternal and neonatal health outcomes. Additionally, the effect size of each individual risk factors and as a whole for GDM, glycaemic patterns during the pregnancy with GDM, and the maternal and neonatal health outcomes including psychosocial wellbeing will be quantified. Furthermore, this study would produce a locally culturally adapted and validated 14-item PBQ and the 19-item MPAS.

This study faces a few challenges. The incidence of some of the CPG-based and potential risk factors is low and may not getting the sufficient numbers for the multivariable regression analysis. The study expands the data source providers by including all the adjacent six public health clinics. This increases the demands of training and supervision of research assistants beside travelling time and coordinating effort between the clinics and university. In case of smaller than expected sample size recruited, we plan the multivariable regression analysis by separate blocks of risk factors. Secondly, the study participants are those receiving care at the public health clinics in the urban areas of Selangor and Putrajaya, thus they may not be representative of the larger Malaysian population. Owing to the Covid-19 pandemic and its effect aftermath, which may delay the data collection processes and a reduction in the number of participants of pregnant women visiting clinics in-person. The decreased in pregnancy in the past year would lower the number of postpartum women with a history of GDM this year. Another limitation of this study may be the quality and accuracy of the antenatal health records. However, we believe this problem is minimal with the many decades long use of the same antenatal records by all the healthcare providers in the public health clinics. The booklet is well-structured with dedicated spaces for the variables to be investigated in this study. We plan clarification and verification strategies to confirm the nature of the risk factors when present but unclear through calling up the women, checking the clinic-based or hospital-based records.

ETHICS AND DISSEMINATION

Risk Benefit Assessment

Participants are not subjected to any medications or treatments during the study period. Also there is no rescue medication or procedures involved. Therefore, there is no direct health risk or might be very least side effects to the participant as this is not an interventional study. There might be a potential risk of fatigue upon completion of the questionnaires. No direct benefit to participants and an honorarium

For peer review only - http://bmjopen.bmj.com/site/about/guidelines.xhtml

of MYR 10.00 will be given. Their participation will provide data that improve understanding in the research topic that may help in the future protocol, guidelines or policymaking.

Ethical consideration

The study will be conducted in compliance with the ethical principles outlined in the Declaration of Helsinki and Malaysian Good Clinical Practice Guideline. Approval from the MREC ethics committee will be obtained. We will clearly explain and state the purpose of this study, also to obtain written consent in Malay or English from all study participants before data collection. Participation is voluntary. They have the right to withdraw at any stage of the research without giving any reason.

Should there be any further amendments to the protocol, other than administrative ones, further approval from the MREC ethics committee will be obtained. Any revisions of documents and amendment to the protocol originally submitted for review, unexpected events during the study period, and new information that may adversely affect the safety of participants and publication will duly be informed to the ethics committee.

Privacy and Confidentiality

Only researchers of this study have access to the participant's data and it will be handled diligently only for this study's purposes. Participant identity will not be revealed as no referencing of participants by name upon presenting the result. The identification number will be used on subject data sheets. All information in this study is confidential. Data from this study will be entered and saved on a dedicated computer that is password protected. Upon completion of the study, softcopy data in the computer will be copied to password protected pen drive and the data in the computer erased. Any hardcopy (including the consent form) and the pen drive will be keep in a locked office of the Principal Investigator's office at UPM and maintained for a minimum of twenty years after the completion of the study. The collected data will be destroyed after that period of storage. Subjects will not be allowed to view their personal study data, as the data will be consolidated into a database. The participants can write to the investigators to request access to the study findings.

Data sharing statement

Data sharing will be considered on a case-by-case basis upon assessment of the proposed study protocol.

Publication Policy

Participants personal information will not be disclosed, thus will not be identified when the findings of this research are published and presented.

Dissemination plan

Research findings will be published in scientific journals, may be presented in scientific conferences, and will be reported and shared with the local health stakeholders.

For peer review only - http://bmjopen.bmj.com/site/about/guidelines.xhtml

Word count: 5716 words from Introduction to Dissemination plan (excluding tables)

Word count: 5218 words from Introduction to Discussion (excluding Ethics & Dissemination, and tables)

CONTRIBUTORSHIP STATEMENT

CBH, NIB, INS, and PY contributed to the study planning and design of the study. The study design was further developed by IP, VR, MB and CBH. PY drafted the manuscript with assistant from CBH and also input from various stages from NMN, ZAS, HH, FP, RZ, SRMA, NBIB, AF and KI. All authors critically reviewed the manuscript and approved the final version as submitted.

COMPETING INTERESTS / CONFLICT OF INTERESTS

None declared.

FUNDING STATEMENT

This study is funded by MYPAIR Grant UK-Malaysia: Joint Partnership Call on Non-Communicable Diseases (Malaysia: JPT.S(BPKl)2000/011/06/05 (27); UK: MR/T018240/1).

DATA SHARING STATEMENT

Data are available upon reasonable request. Data sharing will be considered on a case-by-case basis upon assessment of the proposed study protocol.

ETHICAL APPROVAL

The project has been approved by the Malaysia Research Ethics Committee.

For peer review only

REFERENCES

1. Baz B, Riveline J-P, Gautier J-F. ENDOCRINOLOGY OF PREGNANCY: Gestational diabetes mellitus: definition, aetiological and clinical aspects. European Journal of Endocrinology. 2016 Feb;174(2):R43–51.

2. Buchanan TA, Xiang AH. Gestational diabetes mellitus. J Clin Invest. 2005 Mar 1;115(3):485–91.

3. Cosson E, Carbillon L, Valensi P. High Fasting Plasma Glucose during Early Pregnancy: A Review about Early Gestational Diabetes Mellitus. Journal of Diabetes Research. 2017;2017:1–12.

4. Ganeshan M. Effectiveness of selective risk based screening for Gestational Diabetes (GDM) in Malaysia: A retrospective cohort study based on the National Obstetric Registry (NOR) of Malaysia. 2017;72(1):4.

5. Huhn EA, Rossi SW, Hoesli I, Göbl CS. Controversies in Screening and Diagnostic Criteria for Gestational Diabetes in Early and Late Pregnancy. Front Endocrinol. 2018 Nov 27;9:696.

6. Idris N, Hatikah CC, Murizah M, Rushdan M. Universal versus selective screening for detection of gestational diabetes mellitus in a malaysian population. Malays Fam Physician. 2009;4(2–3):83–7.

7. Plows J, Stanley J, Baker P, Reynolds C, Vickers M. The Pathophysiology of Gestational Diabetes Mellitus. IJMS. 2018 Oct 26;19(11):3342.

8. Nguyen CL, Pham NM, Binns CW, Duong DV, Lee AH. Prevalence of Gestational Diabetes Mellitus in Eastern and Southeastern Asia: A Systematic Review and Meta-Analysis. Journal of Diabetes Research. 2018;2018:1–10.

9. Ravichandran Jeganathan, Shamala Devi Karalasingam. 5th Report of National Obstetrics Registry [Internet]. Jointly published by the National Obstetrics Registry and the Institute Clinical Research (IRC), Ministry of Health Malaysia.; 2016. Available from: http://www.acrm.org.my/nor/

10. Kampan N, Azman H, Hafiz I, Mohammad H, Yee CS, Ghani NAA, et al. OUTCOME OF PREGNANCY AMONG MALAYSIAN WOMEN WITH DIABETES MELLITUS - A SINGLE CENTRE EXPERIENCE. 2013;13:10.

11. Logakodie S, Azahadi O, Fuziah P, Norizzati B, Tan SF, Zienna Z, et al. Gestational diabetes mellitus: The prevalence, associated factors and foeto-maternal outcome of women attending antenatal care. Malays Fam Physician. 2017;12(2):9–17.

12. Lee KW, Ching SM, Hoo FK, Ramachandran V, Chong SC, Tusimin M, et al. Neonatal outcomes and its association among gestational diabetes mellitus with and without depression, anxiety and stress symptoms in Malaysia: A cross-sectional study. Midwifery. 2020 Feb;81:102586.

13. Black MH, Sacks DA, Xiang AH, Lawrence JM. Clinical Outcomes of Pregnancies Complicated by Mild Gestational Diabetes Mellitus Differ by Combinations of Abnormal Oral Glucose Tolerance Test Values. Diabetes Care. 2010 Dec 1;33(12):2524–30.

For peer review only - http://bmjopen.bmj.com/site/about/guidelines.xhtml

14. Feng H, Zhu W-W, Yang H-X, Wei Y-M, Wang C, Su R-N, et al. Relationship between Oral Glucose Tolerance Test Characteristics and Adverse Pregnancy Outcomes among Women with Gestational Diabetes Mellitus: Chinese Medical Journal. 2017 May;130(9):1012–8.

15. de Veciana M, Major CA, Morgan MA, Asrat T, Toohey JS, Lien JM, et al. Postprandial versus preprandial blood glucose monitoring in women with gestational diabetes mellitus requiring insulin therapy. N Engl J Med. 1995 Nov 9;333(19):1237–41.

16. Riskin-Mashiah S, Younes G, Damti A, Auslender R. First-Trimester Fasting Hyperglycemia and Adverse Pregnancy Outcomes. Diabetes Care. 2009 Sep 1;32(9):1639–43.

17. Riskin-Mashiah S, Damti A, Younes G, Auslender R. First trimester fasting hyperglycemia as a predictor for the development of gestational diabetes mellitus. European Journal of Obstetrics & Gynecology and Reproductive Biology. 2010 Oct;152(2):163–7.

18. Zhu W -w., Yang H -x., Wei Y -m., Yan J, Wang Z -l., Li X -l., et al. Evaluation of the Value of Fasting Plasma Glucose in the First Prenatal Visit to Diagnose Gestational Diabetes Mellitus in China. Diabetes Care. 2013 Mar 1;36(3):586–90.

19. Benhalima K, Mathieu C, Damm P, Van Assche A, Devlieger R, Desoye G, et al. A proposal for the use of uniform diagnostic criteria for gestational diabetes in Europe: an opinion paper by the European Board & College of Obstetrics and Gynaecology (EBCOG). Diabetologia. 2015 Jul;58(7):1422–9.

20. Holt H. NICE's latest guidelines on diabetes in pregnancy: getting the balance right. Pract Diab. 2015 Apr;32(3):87–88a.

21. S. S, M. S, R. P, P. K, P. S, Reddy R. HbA1c level in last trimester pregnancy in predicting macrosomia and hypoglycemia in neonate. Int J Contemp Pediatr. 2016;1334–8.

22. Arumugam K, Majeed NA. Glycated haemoglobin is a good predictor of neonatal hypoglycaemia in pregnancies complicated by diabetes. 2011;4.

23. Ministry Of Health. CPG Diabetes in Pregnancy.pdf. Malaysia Health Technology Assessment Section (MaHTAS)Medical Development Division, Ministry of Health Malaysia; 2017.

24. Lee KW, Ching SM, Ramachandran V, Yee A, Hoo FK, Chia YC, et al. Prevalence and risk factors of gestational diabetes mellitus in Asia: a systematic review and meta-analysis. BMC Pregnancy Childbirth. 2018 Dec;18(1):494.

25. Agarwal MM, Punnose J, Dhatt GS. Gestational diabetes: problems associated with the oral glucose tolerance test. Diabetes Research and Clinical Practice. 2004 Jan;63(1):73–4.

26. Nielsen KK, Courten M de, Kapur A. The urgent need for universally applicable simple screening procedures and diagnostic criteria for gestational diabetes mellitus – lessons from projects funded by the World Diabetes Foundation. Global Health Action. 2012 Dec;5(1):17277.

27. Putoto G, Somigliana E, Olivo F, Ponte S, Koroma MM, Citterio F, et al. A simplified diagnostic work-up for the detection of gestational diabetes mellitus in low resources settings: achievements and challenges. Arch Gynecol Obstet. 2020 Nov;302(5):1127–34.

For peer review only - http://bmjopen.bmj.com/site/about/guidelines.xhtml

28. Yong HY, Mohd Shariff Z, Mohd Yusof BN, Rejali Z, Tee YYS, Bindels J, et al. Independent and combined effects of age, body mass index and gestational weight gain on the risk of gestational diabetes mellitus. Sci Rep. 2020 Dec;10(1):8486.

29. Bar-Zeev Y, Haile ZT, Chertok IA. Association Between Prenatal Smoking and Gestational Diabetes Mellitus. Obstetrics & Gynecology. 2020 Jan;135(1):91–9.

30. Dashti S, Abdul latiff latiffah, Abdul Hamid H, Mohamad Saini S, Shah Abu Bakar A, Binti Sabri NAI, et al. Prevalence of Polycystic Ovary Syndrome among Malaysian Female University Staff. JMRH [Internet]. 2019 Jan [cited 2020 Sep 30];7(1). Available from: https://doi.org/10.22038/jmrh.2018.30370.1329

31. Zhu W-W, Yang H-X, Wang C, Su R-N, Feng H, Kapur A. High Prevalence of Gestational Diabetes Mellitus in Beijing: Effect of Maternal Birth Weight and Other Risk Factors. Chinese Medical Journal. 2017 May;130(9):1019–25.

32. Corrado F, Caputo F, Facciolà G, Mancuso A. Gestational Glucose Intolerance in Multiple Pregnancy. Diabetes Care. 2003 May 1;26(5):1646–1646.

33. Hager M, Ott J, Castillo DM, Springer S, Seemann R, Pils S. Prevalence of Gestational Diabetes in Triplet Pregnancies: A Retrospective Cohort Study and Meta-Analysis. J Clin Med [Internet]. 2020 May 18 [cited 2020 Sep 24];9(5). Available from: https://www.ncbi.nlm.nih.gov/pmc/articles/PMC7290297/

34. Bao W, Michels KB, Tobias DK, Li S, Chavarro JE, Gaskins AJ, et al. Parental smoking during pregnancy and the risk of gestational diabetes in the daughter. Int J Epidemiol. 2016 Feb;45(1):160–9.

35. Terry PD, Weiderpass E, Ostenson C-G, Cnattingius S. Cigarette Smoking and the Risk of Gestational and Pregestational Diabetes in Two Consecutive Pregnancies. Diabetes Care. 2003 Nov 1;26(11):2994–8.

36. Borji M, Shahbazi F, Nariman S, Otaghi M, Safari S. Investigating the Relationship Between Mother-Child Bonding and Maternal Mental Health. J Compr Ped [Internet]. 2018 Feb 25 [cited 2021 Jan 5];9(1). Available from: https://sites.kowsarpub.com/jcp/articles/14014.html

37. Marchetti D, Carrozzino D, Fraticelli F, Fulcheri M, Vitacolonna E. Quality of Life in Women with Gestational Diabetes Mellitus: A Systematic Review. Journal of Diabetes Research. 2017;2017:1–12.

38. Bień A, Rzońca E, Kańczugowska A, Iwanowicz-Palus G. Factors Affecting the Quality of Life and the Illness Acceptance of Pregnant Women with Diabetes. IJERPH. 2015 Dec 22;13(1):68.

39. Dalfrà MG, Nicolucci A, Bisson T, Bonsembiante B, Lapolla A. Quality of life in pregnancy and post-partum: a study in diabetic patients. Qual Life Res. 2012 Mar;21(2):291–8.

40. Trutnovsky G, Panzitt T, Magnet E, Stern C, Lang U, Dorfer M. Gestational diabetes: women's concerns, mood state, quality of life and treatment satisfaction. The Journal of Maternal-Fetal & Neonatal Medicine. 2012 Nov;25(11):2464–6.

41. Mishra S, Shetty A, Rao CR, Nayak S, Kamath A. Effect of maternal perceived stress during pregnancy on gestational diabetes mellitus risk: A prospective case-control study. Diabetes & Metabolic Syndrome: Clinical Research & Reviews. 2020 Sep;14(5):1163–9.

For peer review only - http://bmjopen.bmj.com/site/about/guidelines.xhtml

42. Wilson CA, Newham J, Rankin J, Ismail K, Simonoff E, Reynolds RM, et al. Is there an increased risk of perinatal mental disorder in women with gestational diabetes? A systematic review and meta-analysis. Diabet Med. 2020 Apr;37(4):602–22.

43. Feldman R, Weller A, Zagoory-Sharon O, Levine A. Evidence for a Neuroendocrinological Foundation of Human Affiliation: Plasma Oxytocin Levels Across Pregnancy and the Postpartum Period Predict Mother-Infant Bonding. Psychol Sci. 2007 Nov;18(11):965–70.

44. Hassan F, Ching SM, Ling PC, Jaffar N, Lee KW. A Clinical Audit of the Process of Care for Women with Gestational Diabetes Mellitus in a Malaysian Public Polyclinic. 2019;8.

45. The International Association of Diabetes in Pregnancy Study Group (IADPSG) Working Group on Outcome Definitions, Feig DS, Corcoy R, Jensen DM, Kautzky-Willer A, Nolan CJ, et al. Diabetes in pregnancy outcomes: A systematic review and proposed codification of definitions: DM in Pregnancy: Outcome Definitions. Diabetes Metab Res Rev. 2015 Oct;31(7):680–90.

46. World Health Organization. The world health organization quality of life (WHOQOL)-BREF [Internet]. WHO; 2004. Available from: https://www.who.int/publications/i/item/WHO-HIS-HSI-Rev.2012.03

47. Suetsugu Y, Honjo S, Ikeda M, Kamibeppu K. The Japanese version of the Postpartum Bonding Questionnaire: Examination of the reliability, validity, and scale structure. Journal of Psychosomatic Research. 2015 Jul;79(1):55–61.

48. Brockington IF, Oates J, George S, Turner D, Vostanis P, Sullivan M, et al. A Screening Questionnaire for mother-infant bonding disorders. Archives of Women's Mental Health. 2001 Mar 15;3(4):133–40.

49. Condon JT, Corkindale CJ. The assessment of parent-to-infant attachment: Development of a self-report questionnaire instrument. Journal of Reproductive and Infant Psychology. 1998 Feb;16(1):57–76.

50. Spitzer RL, Kroenke K, Williams JBW, Löwe B. A Brief Measure for Assessing Generalized Anxiety Disorder: The GAD-7. Arch Intern Med. 2006 May 22;166(10):1092.

51. Löwe B, Kroenke K, Herzog W, Gräfe K. Measuring depression outcome with a brief self-report instrument: sensitivity to change of the Patient Health Questionnaire (PHQ-9). Journal of Affective Disorders. 2004 Jul;81(1):61–6.

52. Cohen, S., Kamarck, T., Mermelstein, R. Perceived Stress Scale (PSS) [Internet]. Whitaker Insiutute; 1994 [cited 2021 Jan 5]. Available from: https://www.mindgarden.com/documents/PerceivedStressScale.pdf

53. Cheung YB, Yeo KK, Chong KJ, Khoo EY, Wee HL. Reliability and Validity of the English-, Chinese- and Malay-Language Versions of the World Health Organization Quality of Life (WHOQOL-BREF) Questionnaire in Singapore. Ann Acad Med Singap. 2017 Dec;46(12):461–9.

54. Siu BW-M, Ip P, Chow HM-T, Kwok SS-P, Li O-L, Koo M-L, et al. Impairment of mother-infant relationship: validation of the Chinese version of Postpartum Bonding Questionnaire. J Nerv Ment Dis. 2010 Mar;198(3):174–9.

For peer review only - http://bmjopen.bmj.com/site/about/guidelines.xhtml

55. American Psychiatric Association. Diagnostic and Statistical Manual of Mental Disorders, (dsM-iV-tr) 4th Edition (2000) [Internet]. 4th edition. American Psychiatric Pub.; 2000. Available from: https://dsm.psychiatryonline.org/doi/abs/10.1176/appi.books.9780890420249.dsm-iv-tr

56. Sidik SM, Arroll B, Goodyear-Smith F. Validation of the GAD-7 (Malay version) among women attending a primary care clinic in Malaysia. J Prim Health Care. 2012 Mar 1;4(1):5–11, A1.

57. Sherina MS, Arroll B, Goodyear-Smith F. Criterion validity of the PHQ-9 (Malay version) in a primary care clinic in Malaysia. Med J Malaysia. 2012 Jun;67(3):309–15.

58. Azah, M.N.N, Shah, M.E.M, Juwita, S., Bahri, I. S, Rushidi, W. M. W. M, Jamil, Y. M. Validation of the Malay Version Brief Patient Health Questionnaire (PHQ-9) among Adult Attending Family Medicine Clinics. International Medical Journal,. 2005;12(4),:259-263.

59. Gillani S. Perceived Stress Scale Psychometric Validation for Malaysian Diabetic Patients. BJPR. 2011 Jan 10;1(4):156–63.

60. Al-Dubai SAR, Alshagga MA, Rampal KG, Sulaiman NA. Factor Structure and Reliability of the Malay Version of the Perceived Stress Scale among Malaysian Medical Students. Malays J Med Sci. 2012 Jul;19(3):43–9.

61. Sandhu SS, Ismail NH, Rampal KG. The Malay Version of the Perceived Stress Scale (PSS)-10 is a Reliable and Valid Measure for Stress among Nurses in Malaysia. Malays J Med Sci. 2015 Nov;22(6):26–31.

62. N. H. Mazlan, A. Ahmad. A Validity Study of Malay-translated Version of Perceived Stress Scale. Psychology [Internet]. 2012; Available from: https://www.semanticscholar.org/paper/A-Validity-Study-of-Malay-translated-Version-of-Mazlan-Ahmad/9d7eae40b2551aeb5586ab2564d87fa8be060151

63. Epstein J, Santo RM, Guillemin F. A review of guidelines for cross-cultural adaptation of questionnaires could not bring out a consensus. Journal of Clinical Epidemiology. 2015 Apr;68(4):435–41.

64. Vet HCW de, editor. Measurement in medicine: a practical guide. Cambridge ; New York: Cambridge University Press; 2011. 338 p. (Practical guides to biostatistics and epidemiology).

65. Shrout PE, Fleiss JL. Intraclass correlations: Uses in assessing rater reliability. Psychological Bulletin. 1979;86(2):420–8.

66. Mokkink LB, Terwee CB, Patrick DL, Alonso J, Stratford PW, Knol DL, et al. The COSMIN checklist for assessing the methodological quality of studies on measurement properties of health status measurement instruments: an international Delphi study. Qual Life Res. 2010 May;19(4):539–49.

67. Terwee CB, Bot SDM, de Boer MR, van der Windt DAWM, Knol DL, Dekker J, et al. Quality criteria were proposed for measurement properties of health status questionnaires. Journal of Clinical Epidemiology. 2007 Jan;60(1):34–42.

68. Petri E, Palagini L, Bacci O, Borri C, Teristi V, Corezzi C, et al. Maternal–foetal attachment independently predicts the quality of maternal–infant bonding and post-partum

For peer review only - http://bmjopen.bmj.com/site/about/guidelines.xhtml

psychopathology. The Journal of Maternal-Fetal & Neonatal Medicine. 2018 Dec 2;31(23):3153–9.

69. van Bussel JCH, Spitz B, Demyttenaere K. Three self-report questionnaires of the early mother-to-infant bond: reliability and validity of the Dutch version of the MPAS, PBQ and MIBS. Arch Womens Ment Health. 2010 Oct;13(5):373–84.

70. Faul F, Erdfelder E, Buchner A, Lang A-G. Statistical power analyses using G*Power 3.1: Tests for correlation and regression analyses. Behavior Research Methods. 2009 Nov;41(4):1149–60.

71. Kalok A, Ong MY, Hasrori A, Chiang KS, Yazim F, Baharuddin S, et al. Correlation between Oral Glucose Tolerance Test Abnormalities and Adverse Pregnancy Outcomes in Gestational Diabetes: A Cross-Sectional Study. IJERPH. 2020 Sep 24;17(19):6990.

72. Katz MH. Setting up a multivariable analysis. In: Multivariable Analysis. Cambridge: Cambridge University Press; 2011. p. 93–117.

73. the INSPIRED research group, Egan AM, Bogdanet D, Griffin TP, Kgosidialwa O, Cervar-Zivkovic M, et al. A core outcome set for studies of gestational diabetes mellitus prevention and treatment. Diabetologia. 2020 Jun;63(6):1120–7.

For peer review only

TABLES

Table 1 The Malaysian Clinical Practice Guideline 2017 recommended risk factors for GDM screening

Clinical Practice Guideline (CPG) Risk Factors;

1. Age ≥ 25 years old
2. Body mass index > 27 kg/m^2
3. Previous history of GDM
4. First degree relative with diabetes mellitus (DM)
5. History of macrosomia (birth weight > 4 kg)
6. Bad obstetric history (unexplained intrauterine death, neural tube defects, cardiac defects and shoulder dystocia)
7. Glycosuria ≥ 2+ on two occasions
8. Current obstetric problems (essential hypertension, pregnancy-induced hypertension (≥ 140/90 mmHg), polyhydramnios and current use of corticosteroids)

Table 2 Potential new risk factors to be screened for GDM diagnosis and health outcomes

Potential Risk Factors;

1. History of PCOS
2. Current multiple pregnancies
3. Active or passive smoking status
4. Miscarriage (before 23rd week) *(previous and most present)*
5. Pre-term Birth (23rd to 36th week +6 days) *(previous and most present)*
6. Gestational weight gain

For peer review only - http://bmjopen.bmj.com/site/about/guidelines.xhtml

Table 3 Description of the questionnaires

Questionnaire	Description	Score range
The World Health Organization Quality of Life: Brief Version (WHOQOL)-BREF Questionnaire[46]	The WHOQOL-BREF measure is an abbreviated 26-item version of the WHOQOL-100 questionnaire and measures 4 domains of quality of life (QoL): physical (7 items), psychological (6 items), social relationship (3 items) and environment (8 items) domains and 2 additional global items focusing on overall QoL.	Four types of 5-point Likert interval scale are used, inquiring 'how much', 'how often', 'how completely', 'how satisfied' or 'how good' the respondent felt in the past 4-weeks, with different response scale distributed across the domains. Three negatively scored items are reversed scored (3, 4 and 26) and scores are summed up for each domain. Domain scores are computed by taking the mean of the scores and multiplied by 4 (and ranged from 4 to 20) to allow for direct comparison with the WHOQOL-100 scores. Higher domain scores indicate higher QoL. Malay version of this questionnaire showed high internal consistency with Cronbach's alpha ranging from 0.82 to 0.89, which is comparable to the English-language version[53] Interclass Correlation Coefficient ranged from 0.58 to 0.69 across domains, indicating good test-retest reliability[53].
The 14-item Postpartum Bonding Questionnaire (PBQ-14)[47,48]	The PBQ measure will be used to assess the mother-infant relationship during the postpartum period, with a total of 14 items which are rated on a six-point Likert scale from 0 (always) to 5 (never) on four subscales indicating impaired bonding, rejection and anger, anxiety about care, and the risk of abuse.	When the statement is reflecting negative emotion, the scoring is reversed. The summed total score ranges from 0 to 70, with low scores indicating good bonding. The PBQ has acceptable reliability and validity and as for its utility specifically in Asian countries, the measure has been previously tested and demonstrated high sensitivity of 83% and specificity of 96%[48,54].
The 19-item Maternal Postnatal Attachment Scale (MPAS-19)[49]	MPAS is a 19-item self-report questionnaire designed to assess maternal emotional response towards her infant during the first year of life. There are three dimensions: 1) Quality of postnatal attachment (quality of the maternal feelings towards the infant as well as maternal confidence and satisfaction in being a mother);	The three dimensions are considered to be independent but they can be combined to obtain a global attachment score (Total postnatal attachment). The scores on the "Quality" subscale range from 9 to 45, while the scores on the "Pleasure in interaction" and "Absence of hostility" subscales range from 5 to 25. Scores on the global attachment scale range from 19 to 95. Higher scores are generally indicative of stronger attachment but a specific cut-off is not provided[49].

For peer review only - http://bmjopen.bmj.com/site/about/guidelines.xhtml

	2) Absence of hostility (lack of resentment and negative feelings towards the infant) and 3) Pleasure in interaction (desire for proximity and interaction with the infant). Responses are scored on 1 (low attachment) to 5 (high attachment)[49].	
7-item Generalized Anxiety Disorder (GAD 7)[50]	The GAD-7 is a 7-item questionnaire measuring the perceived frequency of generalised anxiety symptoms in the past 2-weeks. The items assess the most prominent diagnostic features of GAD[55]. The items include nervousness, excessive worry, and inability to stop worrying.	Restlessness, easy irritation, difficulty relaxing, and fear of something awful happening on response categories 'not at all', 'several days', 'more than half the days' and 'nearly every day' scored 0,1,2 and 3, respectively. The summed total score ranges from 0 to 21, with higher scores indicating more severe symptoms of anxiety. The Malay version of this questionnaire was found to be valid and reliable measure in women in Malaysia, with high sensitivity of 76% and a specificity of 94%[56].
Patient Health Questionnaire (PHQ-9)[51]	Nine items refer to symptoms experienced by patients during the two weeks prior to answering the questionnaire in making diagnosis and assessing severity of depression.	Scores range from 0 to 27, as each of the nine items is scored from 0 (not at all) to 3 (nearly every day). PHQ-9 scores of 5, 10, 15, and 20 represents mild, moderate, moderately severe, and severe depression, respectively. This questionnaire was found to be a valid and reliable instrument to measure depression, with high sensitivity 87% and specificity of 82% in Malaysia[57]. Good internal reliability with a Cronbach's alpha of 0.67 and test-retest reliability of 0.73 were also demonstrated in this population[58].
The Perceived Stress Scale (PSS)[52]	The PSS measure has 10 items on a 5-point Likert scale ranging from 0 (never) to 4 (very often), assessing the perceived stress levels in the past 4-weeks.	The total score is calculated by reversing the responses for the 4 positively stated items (4, 5, 7 and 8) and then summing across all 10 items. The total score can range from 0 to 56, with higher score representing greater perceived levels of stress. This scale was previously utilised in Malaysian diabetic patients[59], medical students[60], working population[61], and female prisoners[62] in Malaysia and showed comparable psychometric properties to the original English version, with Cronbach's alpha from 0.63 to 0.85 and high test-retest reliability of r =.72.

For peer review only - http://bmjopen.bmj.com/site/about/guidelines.xhtml

FIGURE LEGEND / CAPTION

Figure 1: Risk factors for the diagnosis of gestational diabetes mellitus in different trimester and their relation to maternal and neonatal outcomes

Figure 2: Overview of the procedure in data collection

For peer review only

LIST OF ABBREVIATIONS

BMI	Body Mass Index
CPG	Clinical Practice Guideline
DM	Diabetes Mellitus
FPG	Fasting Plasma Glucose
GAD-7	7-item Generalized Anxiety Disorder
GDM	Gestational Diabetes Mellitus
HBA1C	Glycosylated hemoglobin A1c
2-HPP	2-Hour Post-Prandial
IADPSG	International Association of Diabetes and Pregnancy Study Group
LGA	Large for Gestational Age
MREC	Malaysia Research Ethics Committee
NMRR	National Medical Research Registration
MPAS-19	19-item Maternal Postnatal Attachment Scale
OGTT	Oral Glucose Tolerance Test
PBQ-14	14-item Postpartum Bonding Questionnaire
PCA	Principal component analysis
PCOS	Polycystic Ovarian Syndrome
PHQ-9	9- item Patient Health Questionnaire
PPH	Postpartum Haemorrhage
PSS	Perceived Stress Scale
QoL	Quality of Life
SGA	Small for Gestational Age
TSH	Thyroid Stimulating Hormone
T4	Thyroxine
WHOQOL	World Health Organization Quality of Life

For peer review only

For peer review only - http://bmjopen.bmj.com/site/about/guidelines.xhtml

Figure 1: Risk factors for the diagnosis of gestational diabetes mellitus in different trimester and their relation to maternal and neonatal outcomes

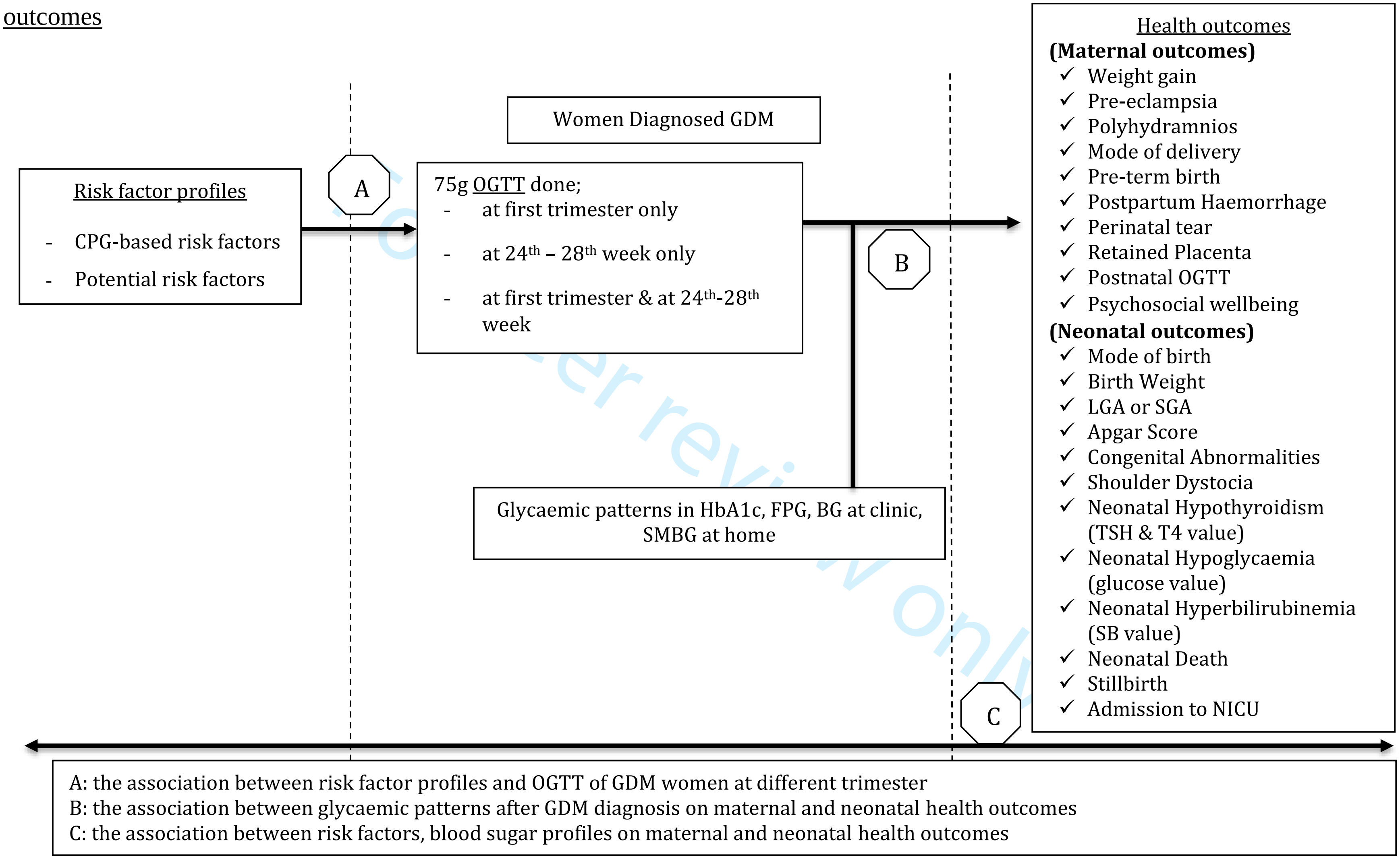

*CPG=Clinical Practice Guidelines, FPG=Fasting Plasma Glucose, BG=Blood Glucose, SMBG=Self-Monitoring Blood Glucose, OGTT=Oral Glucose Tolerance Test, LGA=Large Gestational Age, SGA=Small Gestational Age, TSH=Thyroid Stimulating Hormone, T4=Thyroxine, SB=Serum Bilirubin, NICU=Neonatal Intensive Care Unit

For peer review only

Figure 2: Overview of the procedure in data collection

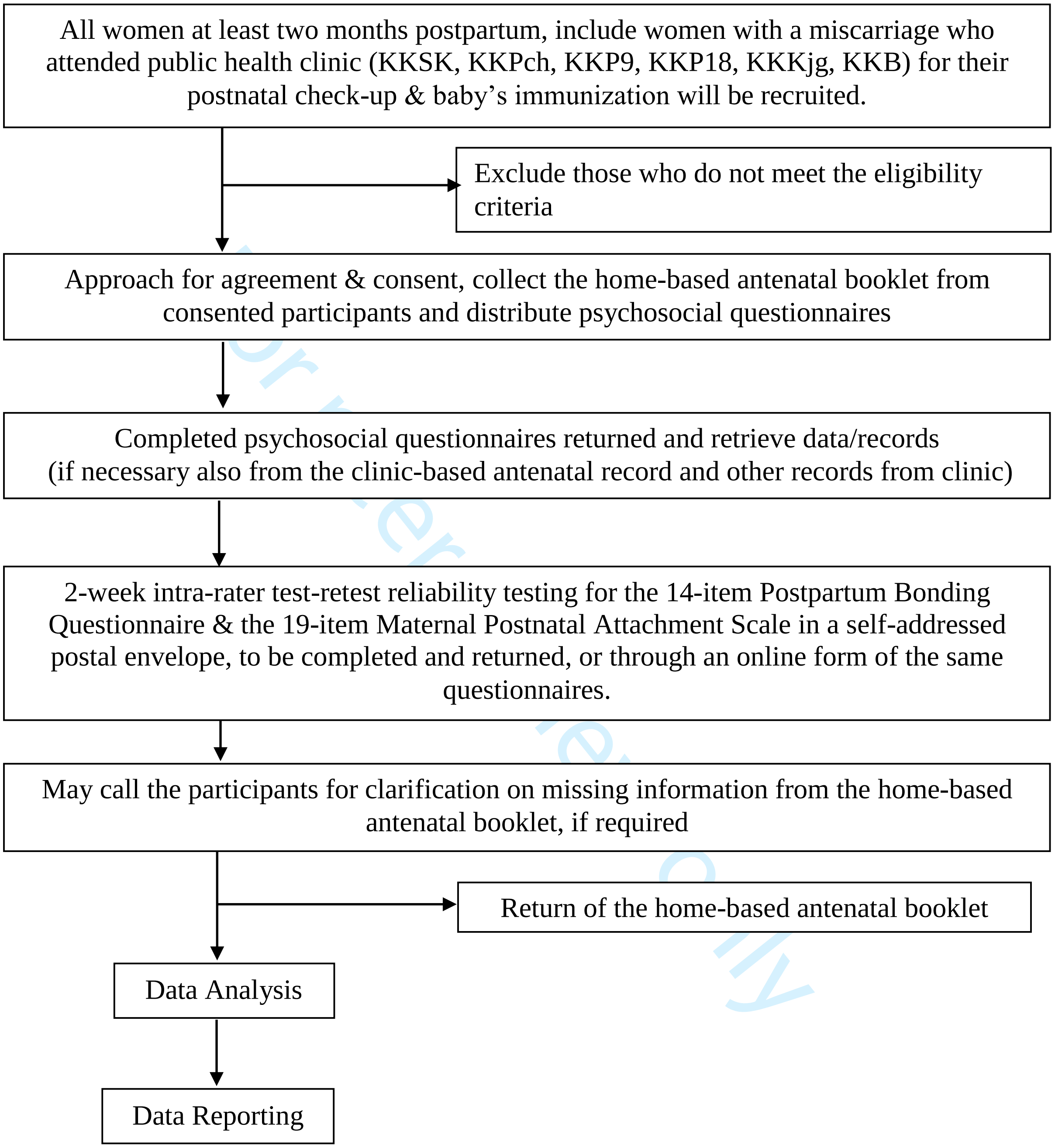

* KKP9= Klinik Kesihatan Precint 9; KKP18= Klinik Kesihatan Precint 18; KKSK= Klinik Kesihatan Seri Kembangan; KKPch= Klinik Kesihatan Puchong; KKKjg= Klinik Kesihatan Kajang; KKB=Klinik Kesihatan Bangi

For peer review only - http://bmjopen.bmj.com/site/about/guidelines.xhtml

STROBE checklist, version1.0, dated 12th April 2021

	Item No		Recommendation	Page No
**Title and abstract**	1		(*a*) Indicate the study's design with a commonly used term in the title or the abstract	1-3
			(*b*) Provide in the abstract an informative and balanced summary of what was done and what was found	3
		**Introduction**		
Background/rationale	2		Explain the scientific background and rationale for the investigation being reported	4-7
Objectives	3		State specific objectives, including any prespecified hypotheses	8
		**Methods**		
Study design	4		Present key elements of study design early in the paper	7-8
Setting	5		Describe the setting, locations, and relevant dates, including periods of recruitment, exposure, follow-up, and data collection	8
Participants	6		(*a*) *Cohort study*—Give the eligibility criteria, and the sources and methods of selection of participants. Describe methods of follow-up<br>*Case-control study*—Give the eligibility criteria, and the sources and methods of case ascertainment and control selection. Give the rationale for the choice of cases and controls<br>*Cross-sectional study*—Give the eligibility criteria, and the sources and methods of selection of participants	na
			(*b*) *Cohort study*—For matched studies, give matching criteria and number of exposed and unexposed<br>*Case-control study*—For matched studies, give matching criteria and the number of controls per case	8
Variables	7		Clearly define all outcomes, exposures, predictors, potential confounders, and effect modifiers. Give diagnostic criteria, if applicable	8-9
Data sources/ measurement	8*		For each variable of interest, give sources of data and details of methods of assessment (measurement). Describe comparability of assessment methods if there is more than one group	8-10
Bias	9		Describe any efforts to address potential sources of bias	na
Study size	10		Explain how the study size was arrived at	11
Quantitative variables	11		Explain how quantitative variables were handled in the analyses. If applicable, describe which groupings were chosen and why	11
Statistical methods	12		(*a*) Describe all statistical methods, including those used to control for confounding	11-13
			(*b*) Describe any methods used to examine subgroups and interactions	11-13
			(*c*) Explain how missing data were addressed	11
			(*d*) *Cohort study*—If applicable, explain how loss to follow-up was addressed<br>*Case-control study*—If applicable, explain how matching of cases and controls was addressed<br>*Cross-sectional study*—If applicable, describe analytical methods taking account of sampling strategy	11-12
			(*e*) Describe any sensitivity analyses	11-13

Continued on next page

For peer review only - http://bmjopen.bmj.com/site/about/guidelines.xhtml

STROBE checklist, version1.0, dated 12th April 2021

**Results**			
Participants	13*	(a) Report numbers of individuals at each stage of study—eg numbers potentially eligible, examined for eligibility, confirmed eligible, included in the study, completing follow-up, and analysed	na
		(b) Give reasons for non-participation at each stage	na
		(c) Consider use of a flow diagram	na
Descriptive data	14*	(a) Give characteristics of study participants (eg demographic, clinical, social) and information on exposures and potential confounders	na
		(b) Indicate number of participants with missing data for each variable of interest	na
		(c) *Cohort study*—Summarise follow-up time (eg, average and total amount)	na
Outcome data	15*	*Cohort study*—Report numbers of outcome events or summary measures over time	na
		Case-control study—Report numbers in each exposure category, or summary measures of exposure	na
		Cross-sectional study—Report numbers of outcome events or summary measures	na
Main results	16	(*a*) Give unadjusted estimates and, if applicable, confounder-adjusted estimates and their precision (eg, 95% confidence interval). Make clear which confounders were adjusted for and why they were included	na
		(*b*) Report category boundaries when continuous variables were categorized	na
		(*c*) If relevant, consider translating estimates of relative risk into absolute risk for a meaningful time period	na
Other analyses	17	Report other analyses done—eg analyses of subgroups and interactions, and sensitivity analyses	na
**Discussion**			
Key results	18	Summarise key results with reference to study objectives	na
Limitations	19	Discuss limitations of the study, taking into account sources of potential bias or imprecision. Discuss both direction and magnitude of any potential bias	13-14
Interpretation	20	Give a cautious overall interpretation of results considering objectives, limitations, multiplicity of analyses, results from similar studies, and other relevant evidence	na
Generalisability	21	Discuss the generalisability (external validity) of the study results	na
**Other information**			
Funding	22	Give the source of funding and the role of the funders for the present study and, if applicable, for the original study on which the present article is based	22

*Give information separately for cases and controls in case-control studies and, if applicable, for exposed and unexposed groups in cohort and cross-sectional studies.

Note: An Explanation and Elaboration article discusses each checklist item and gives methodological background and published examples of transparent reporting. The STROBE checklist is best used in conjunction with this article (freely available on the Web sites of PLoS Medicine at http://www.plosmedicine.org/, Annals of Internal Medicine at http://www.annals.org/, and Epidemiology at http://www.epidem.com/). Information on the STROBE Initiative is available at www.strobe-statement.org.

For peer review only - http://bmjopen.bmj.com/site/about/guidelines.xhtml

BMJ Open

Study protocol on Risk factors for the diagnosis of gestational diabetes mellitus in different trimesters and their relation to maternal and neonatal outcomes (GDM-RIDMAN)

Journal:	*BMJ Open*
Manuscript ID	bmjopen-2021-052554.R1
Article Type:	Protocol
Date Submitted by the Author:	10-Feb-2022

Complete List of Authors:	Yap, Pamela; Universiti Putra Malaysia Fakulti Perubatan dan Sains Kesihatan,<br>Papachristou Nadal, Iliatha; London School of Hygiene and Tropical Medicine, Faculty of Epidemiology and Population Health; King's College London<br>Rysinova, Veronika; King's College London Institute of Psychiatry Psychology and Neuroscience<br>Basri, Nurul Iftida; Universiti Putra Malaysia Fakulti Perubatan dan Sains Kesihatan, Department of Obstetrics and Gynaecology<br>Samsudin, Intan; Universiti Putra Malaysia Fakulti Perubatan dan Sains Kesihatan, Department of Pathology<br>Forbes, Angus; King's College London, Division of Care in Long-term Conditions<br>Noor, Nurain; Hospital Putrajaya Malaysia<br>Supian, Ziti; Klinik Kesihatan Seri Kembangan<br>Hassan, Haslinda ; Klinik Kesihatan Puchong<br>Paimin, Fuziah; Klinik Kesihatan Putrajaya Presint 9<br>Zakaria, Rozita; Klinik Kesihatan Putrajaya Presint 18<br>Mohamed Alias, Siti; Klinik Kesihatan Kajang<br>Ismail Bukhary, Norizzati; Klinik Kesihatan Bangi<br>Benton, Madeleine; King's College London, Department of Psychological Medicine<br>Ismail, Khalida; King's College London, Department of Psychological Medicine<br>Chew, Boon How; Universiti Putra Malaysia, Family Medicine; Hospital Pengajar UPM (UPM Teaching Hospital), Clinical Research Unit

**Primary Subject Heading**:	Diabetes and endocrinology
Secondary Subject Heading:	Obstetrics and gynaecology, Pathology, Mental health
Keywords:	Diabetes in pregnancy < DIABETES & ENDOCRINOLOGY, Clinical chemistry < PATHOLOGY, Depression & mood disorders < PSYCHIATRY

SCHOLARONE™
Manuscripts

Title: Study protocol on Risk factors for the diagnosis of gestational diabetes mellitus in different trimesters and their relation to maternal and neonatal outcomes (GDM-RIDMAN)

Authors:

Pamela Phui Har Yap[1], Iliatha Papachristou[4], Veronika Rysinova[7], Nurul Iftida Basri[2], Intan Nureslyna Samsudin[3], Angus Forbes[5], Nurain Mohd Noor[8], Ziti Akthar Supian[9], Haslinda Hassan[10], Fuziah Paimin[11], Rozita Zakaria[12], Siti Rohani Mohamed Alias[13], Norizzati Bukhary Ismail Bukhary[14], Madeleine Benton[6], Khalida Ismail[6], Boon How Chew[1,15].

Affiliations:

[1]Department of Family Medicine, Faculty of Medicine and Health Sciences, Universiti Putra Malaysia, 43400 Serdang, Selangor, Malaysia.

[2]Department of Obstetrics and Gynaecology, Faculty of Medicine and Health Sciences, Universiti Putra Malaysia, 43400 Serdang, Selangor, Malaysia.

[3]Department of Pathology, Faculty of Medicine and Health Sciences, Universiti Putra Malaysia, 43400 Serdang, Selangor, Malaysia.

[4]King's College London / London School of Hygiene and Tropical Medicine, UK.

[5]Primary and Intermediate Care Section, King's College London.

[6]Department of Psychological Medicine, King's College London.

[7]Institute of Psychiatry, Psychology & Neuroscience, Kings College London.

[8]Hospital Putrajaya, Jalan P9, Presint 7, 62250 Putrajaya, Wilayah Persekutuan Putrajaya, Malaysia.

[9] Klinik Kesihatan Seri Kembangan, Jalan Besar, Taman Muhibbah, 43300 Seri Kembangan, Selangor, Malaysia.

[10]Klinik Kesihatan Puchong Batu 14, 1, Jalan PU 5, Taman Puchong Utama, 47100 Puchong, Selangor, Malaysia.

[11]Klinik Kesihatan Putrajaya Presint 9, 1, Jalan P9e, Presint 9, 62250 Putrajaya, Wilayah Persekutuan Putrajaya, Malaysia.

[12]Klinik Kesihatan Putrajaya Presint 18, Jalan P18c 1, Presint 18, 62150 Putrajaya, Wilayah Persekutuan Putrajaya, Malaysia.

[13]Klinik Kesihatan Kajang, Jalan Semenyih, Bandar Teknologi Kajang, 43500 Kajang, Selangor, Malaysia.

[14]Klinik Kesihatan Bangi, Jalan 3/1, Seksyen 3 Bandar Baru Bangi, 43650 Bandar Baru Bangi, Selangor, Malaysia.

[15]Clinical Research Unit, Hospital Pengajar Universiti Putra Malaysia (HPUPM Teaching Hospital), Persiaran MARDI - UPM, 43400 Serdang, Malaysia

For peer review only

For peer review only - http://bmjopen.bmj.com/site/about/guidelines.xhtml

Corresponding author:

Boon-How Chew, Department of Family Medicine, Faculty of Medicine and Health Sciences, Universiti Putra Malaysia, 43400 Serdang, Selangor, Malaysia. Email: chewboonhow@upm.edu.my.

Word count: 6002/4000 words from Introduction to Dissemination plan (excluding tables)

For peer review only

ABSTRACT (**299**/300 words)

Introduction: Gestational diabetes mellitus (GDM) is often associated with adverse pregnancy outcomes. However, the association of risk factors with GDM diagnosis, maternal and neonatal health outcomes is less established when compared to women without GDM. We will aim to examine the diagnostic accuracy of the conventional and novel risk factors for a GDM diagnosis, and their impact on maternal and neonatal health outcomes.

Methods and analysis: This retrospective cohort and nested case-control study at six public health clinics is based on medical records, and questionnaire survey of women between 2-12 months postpartum. The estimated required sample size is 876 complete records (292 cases, 584 control, at a ratio of 1:2). Oral glucose tolerance test results will be used to identify glucose dysregulation, and maternal and neonatal outcomes include maternal weight gain, pre-eclampsia, polyhydramnios, mode of delivery, pre-term or post-date birth, complications in labour, birth weight, gestational age at birth, Apgar score, congenital anomaly, congenital hypothyroidism, neonatal death or stillbirth, hypoglycaemia and hyperbilirubinemia. Psychosocial measures include the World Health Organization Quality of Life: Brief, mother-infant bonding (14-item Postpartum Bonding Questionnaire and 19-item Maternal Postnatal Attachment Scale), anxiety (7-item Generalized Anxiety Disorder), depression (9-item Patient Health Questionnaire), and stress (Perceived Stress Scale symptoms) questionnaires. The comparative incidences of maternal and neonatal health outcomes, the comparative prevalence of the psychosocial outcomes between women with GDM and without GDM, specificity, sensitivity, positive and negative predictive values of the risk factors, separately and combined, will be reported. All GDM risk factors and outcomes will be modelled using multivariable regression analysis and the receiver operating characteristics curve will be reported.

***Ethics and dissemination*:** This study was approved by the Malaysia Research and Ethics Committee, Ministry of Health Malaysia. Informed consent will be obtained from all participants. Findings will be submitted for publications in scientific journals.

Keywords: gestational diabetes mellitus, risk factors, diagnosis, maternal and neonatal outcomes, antenatal care, postpartum, Malaysia

For peer review only - http://bmjopen.bmj.com/site/about/guidelines.xhtml

Strengths and limitations of this study

- This study provides an opportunity to confirm and explore the conventional risk factors to better predict the diagnosis of GDM.
- Quality of life, mental health and maternal-infant bonding will be assessed during the COVID-19 pandemic.
- Participants are mainly from the urban areas of Selangor and Putrajaya and may not be representative of a larger population.
- The incidence of some of the risk factors may be low and insufficient for inclusion into multivariable regression analysis.
- Recruitment of fewer participants than expected due to the COVID-19 pandemic and the reduced number of pregnancies and postpartum women visiting clinics in-person may introduce challenges in data collection, analysis and interpretation.

For peer review only

For peer review only - http://bmjopen.bmj.com/site/about/guidelines.xhtml

INTRODUCTION

Gestational diabetes mellitus (GDM) is a form of hyperglycaemia, where pregnant women experience glucose intolerance for the first time during the pregnancy[1–3]. Efficient GDM screening and accurate diagnosis allows for early management and treatment which could reduce adverse pregnancy outcomes for both mother and child[4–6].

Asian women are at a higher risk for GDM compared to Caucasian women[7,8]. A systematic review and meta-analysis reported that the prevalence of GDM in Malaysia is in the top five Eastern and Southeast Asian countries where approximately one in nine pregnant women had GDM (11.8%)[8]. The latest National Obstetric Registry 2016-2017 reported the prevalence of GDM with adverse outcomes in Malaysia range from 10.8% to 19.3%[9]. Spontaneous miscarriage and caesarean section are the most frequently reported adverse outcomes in GDM women compared to healthy women, 5.9% vs. 2.6% and 28.5% vs. 18.8%, respectively[10]. Other adverse maternal outcomes include birth trauma, postpartum haemorrhage, pre-eclampsia, and hypertension (≥140/90 mmHg) after the 20th week of gestation with proteinuria[10,11]. Common neonatal adverse outcomes include foetal macrosomia, hypoglycaemia, prematurity, shoulder dystocia, hyperbilirubinemia and admission to intensive care units[11,12]. Prevalence of macrosomic babies and neonatal hypoglycaemia among GDM mothers in Malaysia in 2018 are 4.8%, and 1.7%, respectively[12]. Post-diagnosis postprandial glucose levels are associated with macrosomia and large for gestational age (LGA), premature delivery, gestational hypertension and hyperbilirubinemia[13–15].

At both the local and international level, screening methods and diagnosis of GDM remains debatable. High fasting plasma glucose (FPG) level significantly increases the risk for LGA foetus, primary caesarean section and development of GDM in later pregnancy[16]. Several studies from Israel and Asian population supports FPG ≥6.10 mmol/L at first trimester as the predictor tools for GDM development in later pregnancy[17,18]. Another study in China suggested FPG 6.1-6.9 mmol/L were more accurate and reliable at early pregnancy (before the 24th week) for GDM diagnosis. This differs from the International Association of Diabetes and Pregnancy Study Group (IADPSG) cut-off values of FPG ≥5.1 mmol/L[18,19]. However, this is less well accepted because many perceive FPG ≥5.1 mmol/L to be a false alarm, as when pregnancy advances in a week, FPG level decreases[18]. Glycosylated haemoglobin A1c (HbA1c) is not widely used in practice as the value can be unreliable due to many confounding conditions such as hemoglobinopathy[20]. Although studies have shown the predictive value of HbA1c ≥6.0% of GDM development[5] and adverse neonatal outcomes[10,21,22].

Malaysia practices selective risk-based screening and one-step diagnosis for GDM for early diagnosis and management. All pregnant women will be risk stratified according to the Malaysia 2017 Clinical Practice Guidelines (CPG) on Management of Diabetes in Pregnancy[23]. Women who are perceived to be at risk of GDM will undergo 75g oral glucose tolerance test (OGTT) as soon as the

For peer review only - http://bmjopen.bmj.com/site/about/guidelines.xhtml

next appointment between the 24th to 28th week of gestation[23]. The test requires women to stay fasted from food and drink for at least 8 – 12 hours for FPG and 2 hours after the oral glucose intake. The one-step diagnosis approach of GDM will diagnose GDM when at least any one single abnormal reading is observed at fasting or 2-hour from 75g OGTT[19,23,24]. There are some challenges in full OGTT compliance which include long hours of fasting, drinking of glucose water, vomiting and defaulting the 2-hour post-prandial (2-HPP) plasma glucose test[25–27]. However, there is uncertainty in terms of the state of OGTT compliance and completion rate during the first or second trimester. Similarly, there is a lack of information about the completion rate of OGTT and its association with adverse pregnancy outcomes among pregnant women before the GDM diagnosis[11]. Therefore, adherence of OGTT is equally important during and after pregnancy for GDM women.

The common risk factors of GDM include age ≥25 years old, body mass index >27 kg/m^2, previous history of GDM, first degree relative with diabetes mellitus, history of macrosomia (birth weight >4 kg), bad obstetric history (unexplained intrauterine death, neural tube defects, cardiac defects and shoulder dystocia), glycosuria ≥2+ on two occasions, current obstetric problems (essential hypertension, pregnancy-induced hypertension (≥140/90 mmHg), polyhydramnios and current use of corticosteroids[23] (see Table 1). Other potential risk factors for GDM include polycystic ovarian syndrome (PCOS) (OR 2.33, 95% Cl 1.72-3.17), multiple pregnancies (OR 1.37%, 95% Cl 1.24-1.52), pre-term birth (OR 1.93, 95% Cl 1.21-3.07)[24], maternal gestational weight gain (aOR 3.38%, 95% Cl 1.83-6.24)[28], and maternal smoking status (OR 1.22, 95% Cl 1.08 – 1.38)[29]. The prevalence of PCOS among Malaysian working women age 18 – 49 years was 12.6 %[30] but PCOS is not considered to be a risk factor in the Malaysian CPG. Multiple pregnancies is associated with higher risk for GDM due to a higher weight gain rather than the number of foetus[31]. The rate of multiple pregnancy deliveries among GDM women in Malaysia range approximately from 12% to 30%[32,33]. Many GDM studies have excluded multiple pregnancies in their eligibility criteria, so the risk of multiple pregnancies on GDM is not clear. There is conflicting evidence on smoking and GDM[34,35].

Psychosocial aspects in the postpartum period

There is increasing evidence demonstrating that psychosocial factors, such as depressive symptoms, anxiety, high perceived stress, poor quality of life (QoL) and weakened mother-child bonding play an important role in GDM and adverse neonatal outcomes[12,36–38]. A systematic review demonstrated that GDM respondents consistently showed significantly lower quality of life both short- and long-term compared to healthy pregnant participants[37]. Although the QoL scores significantly improved after pregnancy in healthy women, general health perception in GDM women remained significantly lower[39,40]. The estimated prevalence of depression, anxiety, stress symptoms and poor QoL in GDM women in Malaysia ranges from 10.2% to 39.9%[12].

For peer review only - http://bmjopen.bmj.com/site/about/guidelines.xhtml

Additionally, the odds of GDM were found to be 13-fold higher in women with high stress levels during pregnancy than in women with low stress levels among Indian women[41], suggesting that high perceived stress is a potential risk factor for GDM development. Similarly, a recent systematic review and meta-analysis investigating 62 studies indicated that there was an increased risk of depression and anxiety symptoms around the time of GDM diagnosis and in the postnatal period[42]. Weakened mother-child bonding was previously shown to have a statistically significant association with the mother's depression and anxiety symptoms and these variables also affected mother-child bonding[36]. Mother-child bonding is crucial to the mental growth and development of infants[43]. Given the potential link between GDM and psychosocial factors, investigation and integration of physical and mental health factors in empirical studies and interventions with women experiencing GDM in Malaysia is therefore vitally important and could improve short- and long-term outcomes for women and their children[42].

Conceptual framework

The independent variables are risk factors of GDM including both the CPG-based (Table 1) and the potential risk factors (Table 2) based on the literature. The first dependent variables are the diagnosis of GDM in the first or second trimester as recorded in the maternal records. The second separate dependent variables in this study are the maternal and neonatal health outcomes including the maternal psychological wellbeing. The effect of glycaemic patterns after GDM diagnosis on the maternal and neonatal health outcomes is believed to be present and will be examined. Additionally, we aim to describe the incidences of the maternal and neonatal outcomes during pregnancy and in the postpartum period in women with GDM and without GDM. Figure 1 shows the overall conceptual framework of this study.

There are uncertainties about the differences in risk factor profiles, the antenatal hyperglycaemia patterns, and also the difference on pregnancy outcomes of those who completed the OGTT in the first or second trimester with a GDM diagnosis. Therefore, this study aims to examine and quantify the effects of the current recommended CPG-based risk factors for GDM diagnosis and to compare the risk factor profiles between those associated with GDM diagnoses in the first and second trimester. We will also examine the effects of other potential new risk factors on the actual diagnosis of GDM. Additionally, we will investigate the impacts of all the risk factors and the post-diagnosis blood sugar profiles on the maternal and neonatal outcomes.

For peer review only - http://bmjopen.bmj.com/site/about/guidelines.xhtml

METHOD AND ANALYSIS

This retrospective cohort and nested case-control study will be based on antenatal and postnatal medical records, and will include a questionnaire survey of postpartum women who have delivered within the past 2-12 months. The five study objectives are:

1. To compare the risk factor profiles of women who have undergone OGTT and have been diagnosed with GDM in the first and second trimesters.
2. To describe the occurrences of the maternal health outcomes during pregnancy and in the postpartum period between those with GDM and without GDM.
3. To describe the incidence of the neonatal health outcomes of mothers with GDM and without GDM.
4. To identify and quantify risk factors (including blood sugar profiles) that are associated with any maternal complications.
5. To identify and quantify risk factors (including blood sugar profiles) that are associated with any neonatal complications.

Settings

Data will be collected from six participating public health clinics in Selangor and Putrajaya over approximately 5 months. These clinics are attended by pregnant women of different ethnicities and will provide maternal and childcare services with in-house laboratory services, and availability of GDM registry. The standard care processes in these clinics are that women will be seen by nurses and medical officers or a family medicine specialist when needed for further care. GDM women usually have a follow-up appointment every two weeks for blood sugar level monitoring and pregnancy progress consultation. In post-partum, the appointment ranges from four to six weeks for family planning counselling and a repeat for the OGTT[10,44].

Pre-prandial and post-prandial glucose tests are blood sugar profile tests used for GDM monitoring. A pre-prandial plasma glucose should be ≤5.3 mmol/L[23,45], and post-prandial values at 1-hour and 2-hour are ≤7.8 mmol/L and ≤6.7 mmol/L to be considered as optimal, respectively[23].

Participants

The eligible participants are Malaysian women age ≥18 years old who have undergone OGTT during the last pregnancy, both singleton and multiple pregnancies, with a baby aged at least two months and above *(include pre-term birth between 23 – 37 week of gestation but chronological age at least two months)* or who have had a miscarriage during last pregnancy, receiving most antenatal care at the participating clinics during the last pregnancy and who have returned for a postnatal follow-up at the participating clinics. Malaysian women with pre-existing type 1 or type 2 diabetes and overt diabetes will be excluded from this study.

For peer review only - http://bmjopen.bmj.com/site/about/guidelines.xhtml

Instruments

GDM screening and diagnosis are based on the Malaysian 2017 CPG on Management of Diabetes in Pregnancy[23]. Pregnant women with either one of the abnormal OGTT results, FPG ≥5.1 mmol/L or 2-HPP ≥7.8 mmol/L will be diagnosed as GDM[23]. All data will be retrieved from the antenatal home-based record, and if necessary, from the baby's record book, clinic-based record, GDM registry book, healthcare electronic medical records and laboratory records that are available at each participating centre. If there is insufficient or missing data from the records, we may call the participants for clarification. All variables will be recorded in a structured case record form. All data retrieved will be labelled, stored safely in hard drive and password protected. The independent and dependent variables include the following:

a. Mother's risk factors: age ≥25 years old, at booking BMI >27 kg/m^2, history of GDM, first degree family with diabetes mellitus, previous baby with birth weight >4kg, poor obstetrics medical history (unexplained intrauterine death, congenital abnormalities as such neural tube defects, cardiac defects and shoulder dystocia), glycosuria ≥2+ on two occasions, medical disorders (hypertension ≥140/90 mmHg, polyhydramnios and on corticosteroid medication), multiple pregnancies, smoking status, miscarriage occurring before 23 weeks during the previous or most present pregnancy, and history of PCOS.
b. OGTT result, all blood sugar profiles (blood glucose testing performed at the clinic or home-monitoring) and HbA1c level.
c. Delivery records include:
    i. maternal outcomes (gestational weight gain, pre-eclampsia, polyhydramnios, mode of delivery, gestational age at birth either pre-term or post-date birth and complications in labour including postpartum haemorrhage and perineal tear).
    ii. neonatal outcomes (birth weight, gestational weight at birth either LGA or SGA, Apgar score, congenital anomaly, congenital hypothyroidism from TSH and T4 levels, neonatal death and stillbirth, hypoglycaemia from plasma glucose level, and hyperbilirubinemia from serum bilirubin level).
d. Psychosocial measures include quality of life[46], mother-infant bonding[47–49], anxiety and depression symptoms[50,51], and perceived stress[52] (Table 3).

<u>Cultural adaptation and validation process for questionnaires</u>

The 14-item Postpartum Bonding Questionnaire (PBQ-14) and 19-item Maternal Postnatal Attachment Scale (MPAS-19) will be translated from English to Malay by bilingual translators. Two forward translations from English to Malay will be produced and then another bilingual translator will translate the scale back into English while being blinded to the original English version of the

For peer review only - http://bmjopen.bmj.com/site/about/guidelines.xhtml

questionnaires[53]. The two Malay versions for the scale will be compared with the original and back-translated English version. Based on discussion and consensus by an expert committee, the most appropriate Malay version will be developed and chosen in this study. The expert committee comprises three Malay women with a history of GDM, bilingual (English and Malay) two family physicians, two psychologists and one obstetrician & gynaecologist.

Principal component analysis (PCA) will be used, utilising the Orthogonal rotation (Varimax) to determine the subscales. Preliminary analysis of the PCA output will be made to investigate multicollinearity, sampling adequacy using the Kaiser-Meyer-Olkin statistic and Bartlett's test of sphericity. Internal consistency will be calculated with Cronbach's alpha values for all subscales and 2-week test-retest reliability will be examined with intra-class correlation coefficients (ICC). A Cronbach's alpha value of at least 0.75 indicates good internal consistency of the questionnaire[54]. ICC of at least 0.7 is preferred for a sample size of >50 subjects to estimate the test–retest reliability[55,56].

To check for construct validity (hypothesis-testing validity)[57], associations with GDM status, maternal outcomes (weight gain, pre-eclampsia, polyhydramnios, mode of delivery, pre-term or post-date birth, postpartum haemorrhage, perineal tear, retained placenta, postnatal OGTT result), neonatal outcomes (birth weight, LGA or SGA, Apgar score, congenital abnormalities, shoulder dystocia, neonatal hypothyroidism, neonatal hypoglycaemia, neonatal hyperbilirubinemia, admission to NICU) and the other psychosocial measures (WHOQOL-Bref, GAD-7, PHQ-9, and PSS-10) will be examined. Lastly, PBQ-14, and MPAS-19 will be examined against each other. We hypothesise that maternal feelings of bonding are moderately correlated ($r \geq 0.5$) and associated with maternal mood[58,59]. All statistical analyses will use Statistical Package for Social Sciences (SPSS) version 26.0 (IBM, Chicago, IL) and p value >0.05 will be considered statistically significant.

Sampling process

The study will take place at all six participating health clinics at the same time. All eligible participants will be invited to participate in the study. All women with GDM will be the case. Two control will be sampled for each case. Women in the control group were selected based on the eligibility criteria from the same six health clinics who had undergone OGTT but without a diagnosis of GDM, at about the same period of gestation at the diagnosis of GDM to the case. Women who have consented will leave their home-based health record for the researcher for a period of 1-2 months. They will self-administer the questionnaires (about 30 minutes to complete) online or manually in either Malay or English according to their preference and return it before they leave the clinic. A trained research assistant will be at each participating clinic to facilitate the data collection and to respond to any queries relating to the questionnaire. A researcher (PPHY) will be contactable to answer queries from those who choose to complete the questionnaire online. Participants will answer the online questionnaires at their own convenience to reduce the physical contact and time spent during their clinic visit. A weekly reminder

For peer review only - http://bmjopen.bmj.com/site/about/guidelines.xhtml

in the form of a text message will be sent to the participants who did not complete the questionnaires for two weeks. Every booklet will have a bookmark inserted with a unique code and the booklets will be kept in a safe place in the clinics until collected data retrieval. In return, the participating women will receive a copy of the Participant Information Sheet, signed consent form, and a duplicate bookmark with the same code attached to their health record. The bookmark contains details on the study and the researcher contact information. Participants can request for early return of their home-based record at any time during the study period, and it will be returned to them immediately through the clinic or registered mail. At the end of the study, all home-based records will be returned to each clinic and the women will be informed for collection. They will present the duplicate bookmark for verification to collect their health records. Participants who have completed the questionnaires will receive a token of appreciation when they return to collect their antenatal home-based record.

At each participating clinic, we will extract data from the OGTT records, and only request access to the health records of the consented patients to verify necessary information including the history of miscarriage or pre-term birth. Figure 2 shows the overview of the procedures in data collection.

We will also re-invite at least 50 participants from both case and control group to complete the PBQ-14 and MPAS-19 for the 2-week intra-rater test-retest reliability testing[57]. Assuming a 50% response rate, the first 100 women with and without GDM (total n= 200) will be invited to participate in this reliability test. We will send the PBQ-14 and MPAS-19 online questionnaires to selected participants after 2-week after completing the first.

Sample size estimation

Based on previous studies, the prevalence of GDM with adverse outcomes range from 5-27.9%[4,11]. We use GPower 3.1.9.7[60] with 0.90 power and significance at two-sided α of 0.05 to estimate the smallest difference in maternal or neonatal complication rate between GDM with optimal glycaemic control and suboptimal control to be at 15%, the required sample size is 312 (104 cases, 208 control). For another estimation, the required sample size to estimate 10% (as the lowest possible proportion among all the risk factors) either the history of PCOS with the reported prevalence rate 12.6%[30] or multiple pregnancies with reported incidence of 12-30% among GDM women[32,33] with the power 0.90 and α 0.05 at two tails is 263. Taking into consideration about 10% of incomplete or missing data in the home-based records, the sample size needed is 292 (263/0.90) cases and 584 (526/0.90) control at the ratio of 1:2. Knowing the average number of pregnancies at each of the six participating clinics (200-300 cases and 1500-3000 controls per year) and the required 1:10 ratio of independent variables to numbers of dependent variables to run a multiple logistic regression with 14 independent variables on GDM with any maternal or neonatal complication rate of 40%[61], the study is deemed to be feasible.

For peer review only - http://bmjopen.bmj.com/site/about/guidelines.xhtml

Therefore, we will continue to collect the home-based records from the postpartum women until at least 876 records have been collected.

Data analysis plan

All data analysis will be conducted using SPSS version 26.0 (IBM, Chicago, IL). Data entered will be cleaned and checked for the missing, extreme and suspicious values. These may be verified with the respondents or omitted as missing values. Once the missing data is determined to be missing at random, multiple imputations with ten runs may be conducted to replace the missing data. The complete case analysis will be conducted if the sample size achieves 789 at a minimum.

We will use a descriptive analysis to summarize the sociodemographic data and clinical variables according to the diagnosis of GDM during the first and second trimester at 24th to 28th weeks of gestation. All glycaemic biomarkers including blood sugar profiles will be reported in the trimesters according to the outcomes (normal vs. adverse). Comparisons of mean levels for continuous variables will be analysed using Student's t-test and Chi-square test for categorical variables. The equivalent non-parametric tests will be used for data with non-normal distribution. We will report the proportion of postnatal women who had completed OGTT in the first trimester as well as those who completed OGTT in the second trimester and among each of this group the proportions offered OGTT twice or more (when they need to repeat the test).

To achieve the first objective, we will calculate the proportion of risk factors that are identified according to the trimester when GDM diagnosis is made. We will compare the risk factors profile of women completed OGTT and diagnosed GDM at first trimester to those who have undergone the OGTT but without GDM. A similar analysis will be conducted to compare the risk factor profiles of women with and without GDM who were offered OGTT during the second trimester and not in the first trimester. We will report the specificity, sensitivity, positive and negative predictive values of the CPG-based risk factors and to examine the novel risk factors, separately and combined, for a GDM diagnosis in the first and second trimester, respectively. Additionally, we will compare the risk factors profile of women diagnosed with GDM at the first and second trimester. We will model the risk factor profiles that best predict the GDM diagnosis at the first and second trimester, respectively, using multiple logistic regression. The discrimination ability of the multiple logistic model consisting of the risk factors for GDM will be estimated with the area under the receiver operating characteristics curve with 95% confidence interval.

We will report the comparative incidences of the maternal health outcomes during pregnancy and labour, and the comparative prevalence of the psychosocial outcomes in the postpartum period between the women with GDM and without GDM (second objective). Similarly, we will report the comparative incidences of the neonatal outcomes between the women with GDM and without GDM (third objective).

For peer review only - http://bmjopen.bmj.com/site/about/guidelines.xhtml

The fourth objective can be achieved by comparing the adjusted R^2 values of multivariable logistic regression models consisting of the CPG-based risk factors and models with the added new potential risk factors for GDM diagnosis, including the documented glycaemic biomarkers on the outcome of maternal complications as a whole or by each complication. If sample size allows, this analysis will be conducted separately for GDM diagnosed at first and second trimester to examine its effect on the maternal complications.

Analysis step to measure the fifth objective is similar to the fourth objective by comparing the adjusted R^2 values of multivariable logistic regression models consisting of the CPG-based risk factors and models with the added new potential risk factors for GDM diagnosis, including documented glycaemic biomarkers on neonatal health outcomes.

The current risk factors for GDM will be assessed univariably and multivariably of their effect on the diagnosis of GDM at first and second trimester (24th to 28th weeks) using the R^2 and adjusted R^2. The factors from the demographic and clinical variables on GDM diagnosis will be estimated in univariable logistic regression analyses. Any of this factor with a P-value <0.20 from will be included in the multiple logistics regression analysis[62]. The analyses may be conducted by blocks of risk factors such as sociodemographic information (age, BMI and smoking), family history and past medical history, current antenatal medical problems (glycosuria, obstetric medical conditions and weight gain) if sample size is less than desirable. Multicollinearity between any independent variables will be checked using correlation matrix and standard errors (SE) of each variable[63]. Any two variables correlated > 0.9 or/and the variable has a SE > 5.0 will indicate the presence of multicolinearity. In the presence of multicollinearity, the variable with largest SE and less critical or essential from clinical perspectives will be excluded. This process will continue until the magnitude of the SEs for all the variables hover around 0.001 - 5.0. All final models, Q-Q plots for normality, the residual plots for linearity and homogeneity assumptions and model fitting will be checked. Same statistical strategy may be conducted to model the independent predictors on the maternal and neonatal outcomes. The maternal outcomes (abnormal gestational weight gain, pre-eclampsia, polyhydramnios, abnormal modes of delivery, gestational age at birth either pre-term or post-date birth and complication in labour includes postpartum haemorrhage and perineal tear) will be combined as a whole or separately if sample size for any of the outcome allows, and the maternal psychosocial wellbeing measures will be analysed separately. Similarly, if sample size allows, we will analyse for the neonatal outcomes (birth weight, abnormal gestational weight at birth, poor Apgar score, congenital anomaly, congenital hypothyroidism, neonatal death, stillbirth, hypoglycaemia, and hyperbilirubinemia). Confounding factors will be assessed in multiple logistic regression modelling on maternal and neonatal outcomes, and maternal psychosocial wellbeing measures. A confounding factor is present when it changes the odd ratio of GDM on the outcome by a magnitude of > 10%[64]. This will be done to verify the variables

For peer review only - http://bmjopen.bmj.com/site/about/guidelines.xhtml

included in the final models of the maternal and neonatal outcomes, and maternal psychosocial wellbeing measures.

Strengths and Limitations

This retrospective cohort study incorporates a nested case-control design, providing an opportunity to confirm and explore the conventional CPG-based and potential novel risk factors to better predict the diagnosis of GDM, either at the first or/and second trimester. This is one of the core outcomes to be included in GDM prevention and treatment research[65]. Additionally, we will also examine multiple pregnancies on diagnosis of GDM, which was often excluded from earlier studies. The risk profiles that best predict the diagnosis of GDM will be modelled and examined for their impacts on the maternal and neonatal health outcomes. This evidence is imperative to improve the identification of at-risk women and earlier treatment for GDM. This study would help healthcare practitioners and women with GDM to better understand the effect of both the currently 'recommended' and potentially 'new' important risk factors of GDM, patterns of glycaemic control and their association with health outcomes in both the women and neonates. This is potentially impactful on the decision rule of the existing practice. As this study will be assessing the level of OGTT completion, it will determine whether a delay in the completion of OGTT is one of the possible causes of a delayed GDM diagnosis and treatment, and the risk profiles of women and their association with any maternal and neonatal health outcomes will be identified. Additionally, the effect of each individual risk factor and as a whole on GDM, glycaemic patterns during pregnancy, and the maternal and neonatal health outcomes including psychosocial wellbeing will be quantified. Furthermore, this study would also validate locally and culturally adapted 14-item PBQ and the 19-item MPAS.

This study faces a few limitations. The incidence of some of the CPG-based and potential novel risk factors may be low and insufficient in numbers for the multivariable regression analysis. To overcome this, the study has included all the adjacent six public health clinics. Consequently, this has also increased the demands of training and supervision of research assistants, travelling time and coordinating effort. This will be taken care of by having a written study manual, a site visit to the participating clinics before the start of recruitment, and regular contact with all the research assistants until the end of recruitment. In the case of a smaller than expected sample size being recruited, multivariable regression analysis by separate blocks of risk factors will be conducted. Secondly, the six public health clinics are situated in the urban areas of Selangor and Putrajaya, thus the study participants may not be representative of the larger Malaysian population. Owing to the COVID-19 pandemic, the effects of the aftermath may delay the data collection processes and cause a reduction in the number of pregnant women visiting the clinics in-person. There may also be a decrease in pregnancy in the past year which would lower the number of postpartum women participants with a history of GDM this year. Another limitation may be the quality and accuracy of the data in the

For peer review only - http://bmjopen.bmj.com/site/about/guidelines.xhtml

antenatal health records. However, we believe this problem is minimal with the long use over decades of the same antenatal records by all the healthcare providers in the public health clinics. The booklet is well-structured with dedicated spaces for the variables to be investigated in this study. We plan clarification and verification strategies to confirm the nature of the risk factors when present with doubts by contacting the participant, checking the clinic-based or/and hospital-based records.

Patient and Public Involvement

This is one of non-experimental studies in the MYGODDESS Project (https://rb.gy/ccztw5) where women with GDM during pregnancy and in postpartum periods will be interviewed on important barriers and facilitators of self-care. Women with a history of GDM are involved in the face and content validity testing of the PBQ-14 and MPAS-19 questionnaires. Their opinions on the study design, conduct, reporting and dissemination of results will be sought at appropriate time during the study.

ETHICS AND DISSEMINATION

Risk Benefit Assessment

Participants are not subjected to any medications or treatments during the study period. No rescue medication or procedures will be involved. Since this is not an interventional study, there is therefore no direct health risk and no side effects for the participant. There might be a potential risk of fatigue upon completion of the questionnaires. No direct benefit to participants. A small token of appreciation will be given to all participants. Their participation will provide data that aims to improve and increase understanding in the research topic that may contribute to future protocol, guidelines or policymaking.

Ethical consideration

The study will be conducted in compliance with the ethical principles outlined in the Declaration of Helsinki and Malaysian Good Clinical Practice Guideline. This study obtained approval from the MREC ethics committee. We will clearly explain, state the purpose of this study, and to obtain written consent in Malay or English from all study participants before data collection. Participation is voluntary. They have the right to withdraw at any stage of the research without giving any reason. Should there be any further amendments to the protocol, other than administrative ones, further approval from the MREC ethics committee will be obtained. Any revisions of documents and amendment to the protocol originally submitted for review, unexpected events during the study period, and new information that may adversely affect the safety of participants and publication will duly be informed to the ethics committee.

Privacy and Confidentiality

Only researchers of this study have access to the participants' data and will be handled diligently only for the purpose of this study. Participant identity will not be revealed as there will be no referencing

For peer review only - http://bmjopen.bmj.com/site/about/guidelines.xhtml

of participants by name upon presenting the result. The identification number will be used on subject data sheets. All information in this study is confidential. Data from this study will be entered and saved on a dedicated computer that is password protected. Upon completion of the study, softcopy data in the computer will be copied to a password protected pen drive and the data in the computer will be erased. Any hardcopy (including the consent form) and the pen drive will be kept in the Principal Investigator's locked office at UPM and maintained for a minimum of twenty years after the completion of the study. The collected data will be destroyed after that period of storage. Subjects will not be allowed to view their personal study data as the data will be consolidated into a database. The participants can write to the investigators to request access to the study findings.

Publication Policy

Participants' personal information will not be disclosed, thus will not be identified when the findings of this research are published and presented.

Dissemination plan

Research findings will be published in scientific journals, may be presented in scientific conferences, and will be reported and shared with the local health stakeholders.

Word count: **6002**/4000 words from Introduction to Dissemination plan (excluding tables)

AUTHORS' CONTRIBUTIONS

CBH, NIB, INS, and PY contributed to the study planning and design of the study. The study design was further developed by IP, VR, MB and CBH. PY drafted the manuscript with assistant from CBH and also input from various stages from NMN, ZAS, HH, FP, RZ, SRMA, NBIB, AF and KI. All authors critically reviewed the manuscript and approved the final version as submitted.

FUNDING STATEMENT

This study is funded by MYPAIR Grant UK-Malaysia: Joint Partnership Call on Non-Communicable Diseases (Malaysia: JPT.S(BPKI)2000/011/06/05 (27); UK: MR/T018240/1).

COMPETING INTERESTS / CONFLICT OF INTERESTS

None declared.

DATA SHARING STATEMENTS

Data sharing will be considered on a case-by-case basis upon assessment of the proposed study protocol.

For peer review only - http://bmjopen.bmj.com/site/about/guidelines.xhtml

REFERENCES

1. Baz B, Riveline J-P, Gautier J-F. ENDOCRINOLOGY OF PREGNANCY: Gestational diabetes mellitus: definition, aetiological and clinical aspects. *European Journal of Endocrinology*. 2016;174(2):R43–51.

2. Buchanan TA, Xiang AH. Gestational diabetes mellitus. *J Clin Invest.* 2005;115(3):485–91.

3. Cosson E, Carbillon L, Valensi P. High Fasting Plasma Glucose during Early Pregnancy: A Review about Early Gestational Diabetes Mellitus. *Journal of Diabetes Research*. 2017;2017:1–12.

4. Ganeshan M. Effectiveness of selective risk based screening for Gestational Diabetes (GDM) in Malaysia: A retrospective cohort study based on the National Obstetric Registry (NOR) of Malaysia. 2017;72(1):4.

5. Huhn EA, Rossi SW, Hoesli I, Göbl CS. Controversies in Screening and Diagnostic Criteria for Gestational Diabetes in Early and Late Pregnancy. *Front Endocrinol*. 2018;9:696.

6. Idris N, Hatikah CC, Murizah M, Rushdan M. Universal versus selective screening for detection of gestational diabetes mellitus in a malaysian population. *Malays Fam Physician.* 2009;4(2–3):83–7.

7. Plows J, Stanley J, Baker P, Reynolds C, Vickers M. The Pathophysiology of Gestational Diabetes Mellitus. *IJMS*. 2018;19(11):3342.

8. Nguyen CL, Pham NM, Binns CW, Duong DV, Lee AH. Prevalence of Gestational Diabetes Mellitus in Eastern and Southeastern Asia: A Systematic Review and Meta-Analysis. *Journal of Diabetes Researc*h. 2018;2018:1–10.

9. Ravichandran Jeganathan, Shamala Devi Karalasingam. 5th Report of National Obstetrics Registry [Internet]. Jointly published by the *National Obstetrics Registry and the Institute Clinical Research (IRC), Ministry of Health Malaysia*.; 2016. Available from: http://www.acrm.org.my/nor/

10. Kampan N, Azman H, Hafiz I, Mohammad H, Yee CS, Ghani NAA, et al. Outcome of pregnancy among malaysian women with diabetes mellitus - a single centre experience. 2013;13:10.

11. Logakodie S, Azahadi O, Fuziah P, Norizzati B, Tan SF, Zienna Z, et al. Gestational diabetes mellitus: The prevalence, associated factors and foeto-maternal outcome of women attending antenatal care. *Malays Fam Physician*. 2017;12(2):9–17.

12. Lee KW, Ching SM, Hoo FK, Ramachandran V, Chong SC, Tusimin M, et al. Neonatal outcomes and its association among gestational diabetes mellitus with and without depression, anxiety and stress symptoms in Malaysia: A cross-sectional study. *Midwifery.* 2020;81:102586.

13. Black MH, Sacks DA, Xiang AH, Lawrence JM. Clinical Outcomes of Pregnancies Complicated by Mild Gestational Diabetes Mellitus Differ by Combinations of Abnormal Oral Glucose Tolerance Test Values. *Diabetes Care*. 2010;33(12):2524–30.

14. Feng H, Zhu W-W, Yang H-X, Wei Y-M, Wang C, Su R-N, et al. Relationship between Oral Glucose Tolerance Test Characteristics and Adverse Pregnancy Outcomes among Women with Gestational Diabetes Mellitus: *Chinese Medical Journal.* 2017;130(9):1012–8.

For peer review only - http://bmjopen.bmj.com/site/about/guidelines.xhtml

15. de Veciana M, Major CA, Morgan MA, Asrat T, Toohey JS, Lien JM, et al. Postprandial versus preprandial blood glucose monitoring in women with gestational diabetes mellitus requiring insulin therapy. *N Engl J Med*. 1995;333(19):1237–41.

16. Riskin-Mashiah S, Younes G, Damti A, Auslender R. First-Trimester Fasting Hyperglycemia and Adverse Pregnancy Outcomes. *Diabetes Care*. 2009;32(9):1639–43.

17. Riskin-Mashiah S, Damti A, Younes G, Auslender R. First trimester fasting hyperglycemia as a predictor for the development of gestational diabetes mellitus. *European Journal of Obstetrics & Gynecology and Reproductive Biology*. 2010;152(2):163–7.

18. Zhu W -w., Yang H -x., Wei Y -m., Yan J, Wang Z -l., Li X -l., et al. Evaluation of the Value of Fasting Plasma Glucose in the First Prenatal Visit to Diagnose Gestational Diabetes Mellitus in China. *Diabetes Care*. 2013;36(3):586–90.

19. Benhalima K, Mathieu C, Damm P, Van Assche A, Devlieger R, Desoye G, et al. A proposal for the use of uniform diagnostic criteria for gestational diabetes in Europe: an opinion paper by the European Board & College of Obstetrics and Gynaecology (EBCOG). *Diabetologia*. 2015;58(7):1422–9.

20. Holt H. NICE's latest guidelines on diabetes in pregnancy: getting the balance right. *Pract Diab*. 2015;32(3):87–88a.

21. S. S, M. S, R. P, P. K, P. S, Reddy R. HbA1c level in last trimester pregnancy in predicting macrosomia and hypoglycemia in neonate. *Int J Contemp Pediatr*. 2016;1334–8.

22. Arumugam K, Majeed NA. Glycated haemoglobin is a good predictor of neonatal hypoglycaemia in pregnancies complicated by diabetes. 2011;4.

23. Ministry Of Health. CPG Diabetes in Pregnancy.pdf. Malaysia Health Technology Assessment Section (MaHTAS)Medical Development Division, Ministry of Health Malaysia; 2017.

24. Lee KW, Ching SM, Ramachandran V, Yee A, Hoo FK, Chia YC, et al. Prevalence and risk factors of gestational diabetes mellitus in Asia: a systematic review and meta-analysis. *BMC Pregnancy Childbirth*. 2018;18(1):494.

25. Agarwal MM, Punnose J, Dhatt GS. Gestational diabetes: problems associated with the oral glucose tolerance test. *Diabetes Research and Clinical Practice*. 2004;63(1):73–4.

26. Nielsen KK, Courten M de, Kapur A. The urgent need for universally applicable simple screening procedures and diagnostic criteria for gestational diabetes mellitus – lessons from projects funded by the World Diabetes Foundation. *Global Health Action*. 2012;5(1):17277.

27. Putoto G, Somigliana E, Olivo F, Ponte S, Koroma MM, Citterio F, et al. A simplified diagnostic work-up for the detection of gestational diabetes mellitus in low resources settings: achievements and challenges. *Arch Gynecol Obstet*. 2020;302(5):1127–34.

28. Yong HY, Mohd Shariff Z, Mohd Yusof BN, Rejali Z, Tee YYS, Bindels J, et al. Independent and combined effects of age, body mass index and gestational weight gain on the risk of gestational diabetes mellitus. *Sci Rep*. 2020;10(1):8486.

29. Bar-Zeev Y, Haile ZT, Chertok IA. Association Between Prenatal Smoking and Gestational Diabetes Mellitus. *Obstetrics & Gynecology*. 2020;135(1):91–9.

For peer review only - http://bmjopen.bmj.com/site/about/guidelines.xhtml

30. Dashti S, Abdul latiff latiffah, Abdul Hamid H, Mohamad Saini S, Shah Abu Bakar A, Binti Sabri NAI, et al. Prevalence of Polycystic Ovary Syndrome among Malaysian Female University Staff. *JMRH* [Internet]. 2019[cited 2020 Sep 30];7(1). Available from: https://doi.org/10.22038/jmrh.2018.30370.1329

31. Zhu W-W, Yang H-X, Wang C, Su R-N, Feng H, Kapur A. High Prevalence of Gestational Diabetes Mellitus in Beijing: Effect of Maternal Birth Weight and Other Risk Factors. *Chinese Medical Journal.* 2017;130(9):1019–25.

32. Corrado F, Caputo F, Facciolà G, Mancuso A. Gestational Glucose Intolerance in Multiple Pregnancy. *Diabetes Care*. 2003;26(5):1646–1646.

33. Hager M, Ott J, Castillo DM, Springer S, Seemann R, Pils S. Prevalence of Gestational Diabetes in Triplet Pregnancies: A Retrospective Cohort Study and Meta-Analysis. *J Clin Med* [Internet]. 2020[cited 2020 Sep 24];9(5). Available from: https://www.ncbi.nlm.nih.gov/pmc/articles/PMC7290297/

34. Bao W, Michels KB, Tobias DK, Li S, Chavarro JE, Gaskins AJ, et al. Parental smoking during pregnancy and the risk of gestational diabetes in the daughter. *Int J Epidemiol.* 2016;45(1):160–9.

35. Terry PD, Weiderpass E, Ostenson C-G, Cnattingius S. Cigarette Smoking and the Risk of Gestational and Pregestational Diabetes in Two Consecutive Pregnancies. *Diabetes Care.* 2003;26(11):2994–8.

36. Borji M, Shahbazi F, Nariman S, Otaghi M, Safari S. Investigating the Relationship Between Mother-Child Bonding and Maternal Mental Health. J Compr Ped [Internet]. 2018 [cited 2021 Jan 5];9(1). Available from: https://sites.kowsarpub.com/jcp/articles/14014.html

37. Marchetti D, Carrozzino D, Fraticelli F, Fulcheri M, Vitacolonna E. Quality of Life in Women with Gestational Diabetes Mellitus: A Systematic Review. *Journal of Diabetes Research.* 2017;2017:1–12.

38. Bień A, Rzońca E, Kańczugowska A, Iwanowicz-Palus G. Factors Affecting the Quality of Life and the Illness Acceptance of Pregnant Women with Diabetes. *IJERPH*. 2015;13(1):68.

39. Dalfrà MG, Nicolucci A, Bisson T, Bonsembiante B, Lapolla A. Quality of life in pregnancy and post-partum: a study in diabetic patients. *Qual Life Res*. 2012;21(2):291–8.

40. Trutnovsky G, Panzitt T, Magnet E, Stern C, Lang U, Dorfer M. Gestational diabetes: women's concerns, mood state, quality of life and treatment satisfaction. *The Journal of Maternal-Fetal & Neonatal Medicine*. 2012;25(11):2464–6.

41. Mishra S, Shetty A, Rao CR, Nayak S, Kamath A. Effect of maternal perceived stress during pregnancy on gestational diabetes mellitus risk: A prospective case-control study. *Diabetes & Metabolic Syndrome: Clinical Research & Reviews.* 2020;14(5):1163–9.

42. Wilson CA, Newham J, Rankin J, Ismail K, Simonoff E, Reynolds RM, et al. Is there an increased risk of perinatal mental disorder in women with gestational diabetes? A systematic review and meta-analysis. *Diabet Med.* 2020;37(4):602–22.

For peer review only - http://bmjopen.bmj.com/site/about/guidelines.xhtml

43. Feldman R, Weller A, Zagoory-Sharon O, Levine A. Evidence for a Neuroendocrinological Foundation of Human Affiliation: Plasma Oxytocin Levels Across Pregnancy and the Postpartum Period Predict Mother-Infant Bonding. *Psychol Sci*. 2007;18(11):965–70.

44. Hassan F, Ching SM, Ling PC, Jaffar N, Lee KW. A Clinical Audit of the Process of Care for Women with Gestational Diabetes Mellitus in a Malaysian Public Polyclinic. *Mal J Med Health Sci*. 2019;15(2): 69-76.

45. The International Association of Diabetes in Pregnancy Study Group (IADPSG) Working Group on Outcome Definitions, Feig DS, Corcoy R, Jensen DM, Kautzky-Willer A, Nolan CJ, et al. Diabetes in pregnancy outcomes: A systematic review and proposed codification of definitions: DM in Pregnancy: Outcome Definitions. *Diabetes Metab Res Rev*. 2015;31(7):680–90.

46. World Health Organization. The world health organization quality of life (WHOQOL)-BREF [Internet]. *WHO*; 2004. Available from: https://www.who.int/publications/i/item/WHO-HIS-HSI-Rev.2012.03

47. Brockington IF, Oates J, George S, Turner D, Vostanis P, Sullivan M, et al. A Screening Questionnaire for mother-infant bonding disorders. *Archives of Women's Mental Health.* 2001;3(4):133–40.

48. Suetsugu Y, Honjo S, Ikeda M, Kamibeppu K. The Japanese version of the Postpartum Bonding Questionnaire: Examination of the reliability, validity, and scale structure. *Journal of Psychosomatic Research*. 2015;79(1):55–61.

49. Condon JT, Corkindale CJ. The assessment of parent-to-infant attachment: Development of a self-report questionnaire instrument. *Journal of Reproductive and Infant Psychology.* 1998;16(1):57–76.

50. Spitzer RL, Kroenke K, Williams JBW, Löwe B. A Brief Measure for Assessing Generalized Anxiety Disorder: The GAD-7. *Arch Intern Med*. 2006;166(10):1092.

51. Löwe B, Kroenke K, Herzog W, Gräfe K. Measuring depression outcome with a brief self-report instrument: sensitivity to change of the Patient Health Questionnaire (PHQ-9). *Journal of Affective Disorders.* 2004;81(1):61–6.

52. Cohen, S., Kamarck, T., Mermelstein, R. Perceived Stress Scale (PSS) [Internet]. Whitaker Instiutute; 1994 [cited 2021 Jan 5]. Available from: https://www.mindgarden.com/documents/PerceivedStressScale.pdf

53. Epstein J, Santo RM, Guillemin F. A review of guidelines for cross-cultural adaptation of questionnaires could not bring out a consensus. *Journal of Clinical Epidemiology.* 2015;68(4):435–41.

54. Vet HCW de, editor. Measurement in medicine: a practical guide. Cambridge ; New York: Cambridge University Press; 2011. 338 p. (Practical guides to biostatistics and epidemiology).

55. Shrout PE, Fleiss JL. Intraclass correlations: Uses in assessing rater reliability. *Psychological Bulletin.* 1979;86(2):420–8.

56. Mokkink LB, Terwee CB, Patrick DL, Alonso J, Stratford PW, Knol DL, et al. The COSMIN checklist for assessing the methodological quality of studies on measurement properties of health

For peer review only - http://bmjopen.bmj.com/site/about/guidelines.xhtml

status measurement instruments: an international Delphi study. *Qual Life Res.* 2010;19(4):539–49.

57. Terwee CB, Bot SDM, de Boer MR, van der Windt DAWM, Knol DL, Dekker J, et al. Quality criteria were proposed for measurement properties of health status questionnaires. *Journal of Clinical Epidemiology*. 2007;60(1):34–42.

58. Petri E, Palagini L, Bacci O, Borri C, Teristi V, Corezzi C, et al. Maternal–foetal attachment independently predicts the quality of maternal–infant bonding and post-partum psychopathology. *The Journal of Maternal-Fetal & Neonatal Medicine*. 2018;31(23):3153–9.

59. van Bussel JCH, Spitz B, Demyttenaere K. Three self-report questionnaires of the early mother-to-infant bond: reliability and validity of the Dutch version of the MPAS, PBQ and MIBS. *Arch Womens Ment Health*. 2010;13(5):373–84.

60. Faul F, Erdfelder E, Buchner A, Lang A-G. Statistical power analyses using G*Power 3.1: Tests for correlation and regression analyses. *Behavior Research Methods.* 2009;41(4):1149–60.

61. Kalok A, Ong MY, Hasrori A, Chiang KS, Yazim F, Baharuddin S, et al. Correlation between Oral Glucose Tolerance Test Abnormalities and Adverse Pregnancy Outcomes in Gestational Diabetes: A Cross-Sectional Study. *IJERPH*. 2020;17(19):6990.

62. Katz MH. Setting up a multivariable analysis. In: Multivariable Analysis. Cambridge: Cambridge University Press; 2011. p. 93–117.

63. Chan YH. Biostatistics 202: logistic regression analysis. *Singapore Med J*. 2004;45(4):149–53.

64. Grobbee DE, Hoes AW. Clinical epidemiology: principles, methods, and applications for clinical research. Second edition. Burlington, Massachusetts: Jones & Bartlett Learning; 2015. 472 p.

65. the INSPIRED research group, Egan AM, Bogdanet D, Griffin TP, Kgosidialwa O, Cervar-Zivkovic M, et al. A core outcome set for studies of gestational diabetes mellitus prevention and treatment. *Diabetologia*. 2020;63(6):1120–7.

66. Cheung YB, Yeo KK, Chong KJ, Khoo EY, Wee HL. Reliability and Validity of the English-, Chinese- and Malay-Language Versions of the World Health Organization Quality of Life (WHOQOL-BREF) Questionnaire in Singapore. *Ann Acad Med Singap*. 2017;46(12):461–9.

67. Siu BW-M, Ip P, Chow HM-T, Kwok SS-P, Li O-L, Koo M-L, et al. Impairment of mother-infant relationship: validation of the Chinese version of Postpartum Bonding Questionnaire. *J Nerv Ment Dis*. 2010;198(3):174–9.

68. American Psychiatric Association. Diagnostic and Statistical Manual of Mental Disorders, (dsM-iV-tr) 4th Edition (2000) [Internet]. 4th edition. *American Psychiatric Pub.*; 2000. Available from: https://dsm.psychiatryonline.org/doi/abs/10.1176/appi.books.9780890420249.dsm-iv-tr

69. Sidik SM, Arroll B, Goodyear-Smith F. Validation of the GAD-7 (Malay version) among women attending a primary care clinic in Malaysia. *J Prim Health Care*. 2012;4(1):5–11, A1.

70. Sherina MS, Arroll B, Goodyear-Smith F. Criterion validity of the PHQ-9 (Malay version) in a primary care clinic in Malaysia. *Med J Malaysia.* 2012;67(3):309–15.

For peer review only - http://bmjopen.bmj.com/site/about/guidelines.xhtml

71. Azah, M.N.N, Shah, M.E.M, Juwita, S., Bahri, I. S, Rushidi, W. M. W. M, Jamil, Y. M. Validation of the Malay Version Brief Patient Health Questionnaire (PHQ-9) among Adult Attending Family Medicine Clinics. *International Medical Journal*,. 2005;12(4),:259-263.

72. Gillani S. Perceived Stress Scale Psychometric Validation for Malaysian Diabetic Patients. *BJPR*. 2011;1(4):156–63.

73. Al-Dubai SAR, Alshagga MA, Rampal KG, Sulaiman NA. Factor Structure and Reliability of the Malay Version of the Perceived Stress Scale among Malaysian Medical Students. *Malays J Med Sci*. 2012;19(3):43–9.

74. Sandhu SS, Ismail NH, Rampal KG. The Malay Version of the Perceived Stress Scale (PSS)-10 is a Reliable and Valid Measure for Stress among Nurses in Malaysia. *Malays J Med Sci.* 2015;22(6):26–31.

75. N. H. Mazlan, A. Ahmad. A Validity Study of Malay-translated Version of Perceived Stress Scale. *Psychology* [Internet]. 2012; Available from: https://www.semanticscholar.org/paper/A-Validity-Study-of-Malay-translated-Version-of-Mazlan-Ahmad/9d7eae40b2551aeb5586ab2564d87fa8be060151

For peer review only

TABLES

Table 1 The Malaysian Clinical Practice Guideline 2017 recommended risk factors for GDM screening

Clinical Practice Guideline (CPG) Risk Factors;<br>1. Age ≥ 25 years old<br>2. Body mass index > 27 kg/m^2<br>3. Previous history of GDM<br>4. First degree relative with diabetes mellitus (DM)<br>5. History of macrosomia (birth weight > 4 kg)<br>6. Bad obstetric history (unexplained intrauterine death, neural tube defects, cardiac defects and shoulder dystocia)<br>7. Glycosuria ≥ 2+ on two occasions<br>8. Current obstetric problems (essential hypertension, pregnancy-induced hypertension (≥ 140/90 mmHg), polyhydramnios and current use of corticosteroids)

Table 2 Potential new risk factors to be screened for GDM diagnosis and health outcomes

Potential Risk Factors;<br>1. History of PCOS<br>2. Current multiple pregnancies<br>3. Active or passive smoking status<br>4. Miscarriage (before 23rd week) *(previous and most present)*<br>5. Pre-term Birth (23rd to 36th week +6 days) *(previous and most present)*<br>6. Gestational weight gain

For peer review only - http://bmjopen.bmj.com/site/about/guidelines.xhtml

Table 3 Description of the questionnaires

Questionnaire	Description	Score range
The World Health Organization Quality of Life: Brief Version (WHOQOL)-BREF Questionnaire[46]	The WHOQOL-BREF measure is an abbreviated 26-item version of the WHOQOL-100 questionnaire and measures 4 domains of quality of life (QoL): physical (7 items), psychological (6 items), social relationship (3 items) and environment (8 items) domains and 2 additional global items focusing on overall QoL.	Four types of 5-point Likert interval scale are used, inquiring 'how much', 'how often', 'how completely', 'how satisfied' or 'how good' the respondent felt in the past 4-weeks, with different response scale distributed across the domains. Three negatively scored items are reversed scored (3, 4 and 26) and scores are summed up for each domain. Domain scores are computed by taking the mean of the scores and multiplied by 4 (and ranged from 4 to 20) to allow for direct comparison with the WHOQOL-100 scores. Higher domain scores indicate higher QoL. Malay version of this questionnaire showed high internal consistency with Cronbach's alpha ranging from 0.82 to 0.89, which is comparable to the English-language version[66] Interclass Correlation Coefficient ranged from 0.58 to 0.69 across domains, indicating good test-retest reliability[66].
The 14-item Postpartum Bonding Questionnaire (PBQ-14)[47,48]	The PBQ measure will be used to assess the mother-infant relationship during the postpartum period, with a total of 14 items which are rated on a six-point Likert scale from 0 (always) to 5 (never) on four subscales indicating impaired bonding, rejection and anger, anxiety about care, and the risk of abuse.	When the statement is reflecting negative emotion, the scoring is reversed. The summed total score ranges from 0 to 70, with low scores indicating good bonding. The PBQ has acceptable reliability and validity and as for its utility specifically in Asian countries, the measure has been previously tested and demonstrated high sensitivity of 83% and specificity of 96%[47,67].
The 19-item Maternal Postnatal Attachment Scale (MPAS-19)[49]	MPAS is a 19-item self-report questionnaire designed to assess maternal emotional response towards her infant during the first year of life. There are three dimensions: 1) Quality of postnatal attachment (quality of the maternal feelings towards the infant as well as maternal confidence and satisfaction in being a mother);	The three dimensions are considered to be independent but they can be combined to obtain a global attachment score (Total postnatal attachment). The scores on the "Quality" subscale range from 9 to 45, while the scores on the "Pleasure in interaction" and "Absence of hostility" subscales range from 5 to 25. Scores on the global attachment scale range from 19 to 95. Higher scores are generally indicative of stronger attachment but a specific cut-off is not provided[49].

For peer review only - http://bmjopen.bmj.com/site/about/guidelines.xhtml

	2) Absence of hostility (lack of resentment and negative feelings towards the infant) and 3) Pleasure in interaction (desire for proximity and interaction with the infant). Responses are scored on 1 (low attachment) to 5 (high attachment)[49].	
7-item Generalized Anxiety Disorder (GAD 7)[50]	The GAD-7 is a 7-item questionnaire measuring the perceived frequency of generalised anxiety symptoms in the past 2-weeks. The items assess the most prominent diagnostic features of GAD[68]. The items include nervousness, excessive worry, and inability to stop worrying.	Restlessness, easy irritation, difficulty relaxing, and fear of something awful happening on response categories 'not at all', 'several days', 'more than half the days' and 'nearly every day' scored 0,1,2 and 3, respectively. The summed total score ranges from 0 to 21, with higher scores indicating more severe symptoms of anxiety. The Malay version of this questionnaire was found to be valid and reliable measure in women in Malaysia, with high sensitivity of 76% and a specificity of 94%[69].
Patient Health Questionnaire (PHQ-9)[51]	Nine items refer to symptoms experienced by patients during the two weeks prior to answering the questionnaire in making diagnosis and assessing severity of depression.	Scores range from 0 to 27, as each of the nine items is scored from 0 (not at all) to 3 (nearly every day). PHQ-9 scores of 5, 10, 15, and 20 represents mild, moderate, moderately severe, and severe depression, respectively. This questionnaire was found to be a valid and reliable instrument to measure depression, with high sensitivity 87% and specificity of 82% in Malaysia[70]. Good internal reliability with a Cronbach's alpha of 0.67 and test-retest reliability of 0.73 were also demonstrated in this population[71].
The Perceived Stress Scale (PSS)[52]	The PSS measure has 10 items on a 5-point Likert scale ranging from 0 (never) to 4 (very often), assessing the perceived stress levels in the past 4-weeks.	The total score is calculated by reversing the responses for the 4 positively stated items (4, 5, 7 and 8) and then summing across all 10 items. The total score can range from 0 to 56, with higher score representing greater perceived levels of stress. This scale was previously utilised in Malaysian diabetic patients[72], medical students[73], working population[74], and female prisoners[75] in Malaysia and showed comparable psychometric properties to the original English version, with Cronbach's alpha from 0.63 to 0.85 and high test-retest reliability of r =.72.

FIGURE LEGEND / CAPTION

Figure 1: Risk factors for the diagnosis of gestational diabetes mellitus in different trimester and their relation to maternal and neonatal outcomes

Figure 2: Overview of the procedure in data collection

For peer review only

Figure 1: Risk factors for the diagnosis of gestational diabetes mellitus in different trimester and their relation to maternal and neonatal outcomes

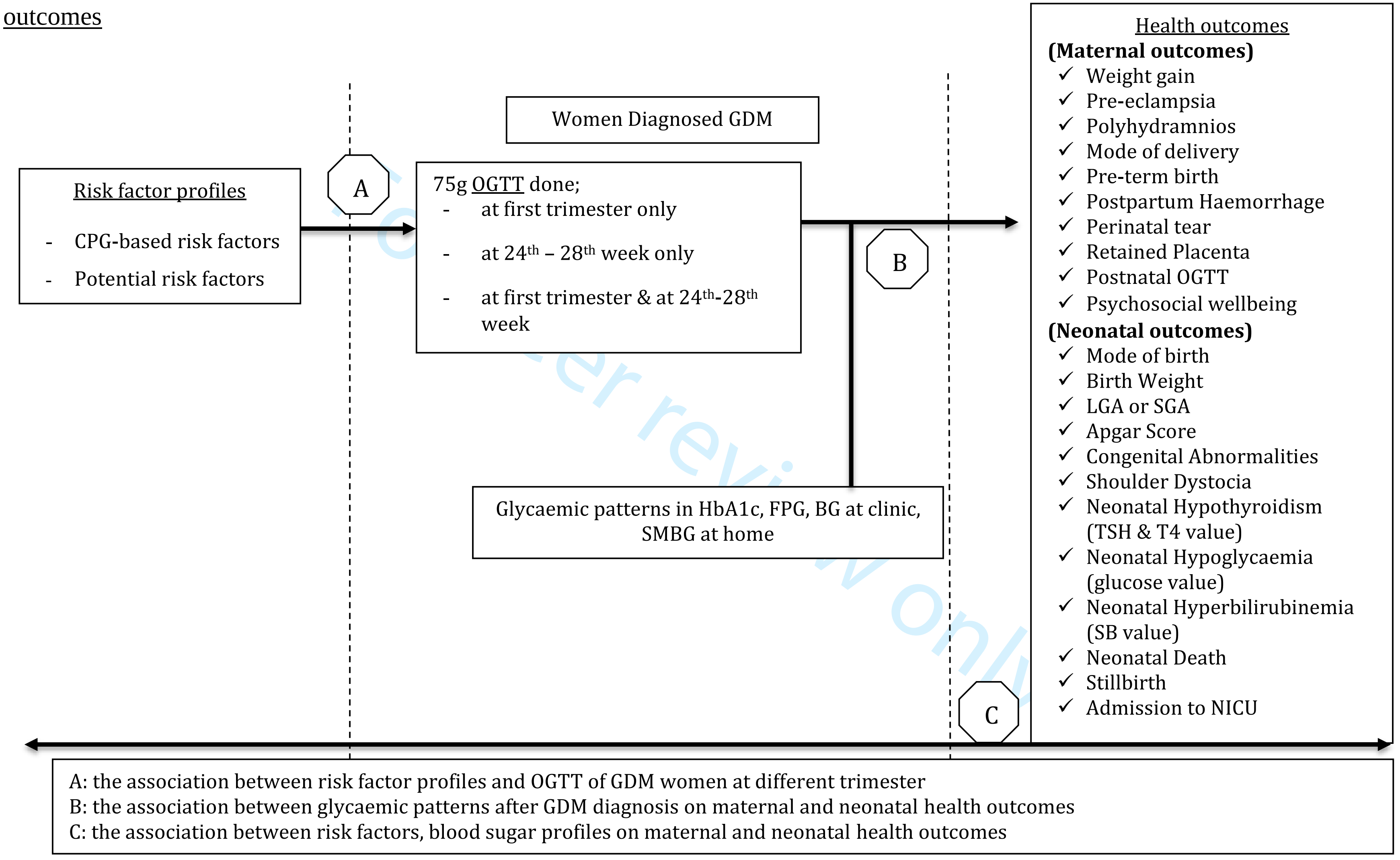

*CPG=Clinical Practice Guidelines, FPG=Fasting Plasma Glucose, BG=Blood Glucose, SMBG=Self-Monitoring Blood Glucose, OGTT=Oral Glucose Tolerance Test, LGA=Large Gestational Age, SGA=Small Gestational Age, TSH=Thyroid Stimulating Hormone, T4=Thyroxine, SB=Serum Bilirubin, NICU=Neonatal Intensive Care Unit

For peer review only - http://bmjopen.bmj.com/site/about/guidelines.xhtml

Figure 2: Overview of the procedure in data collection

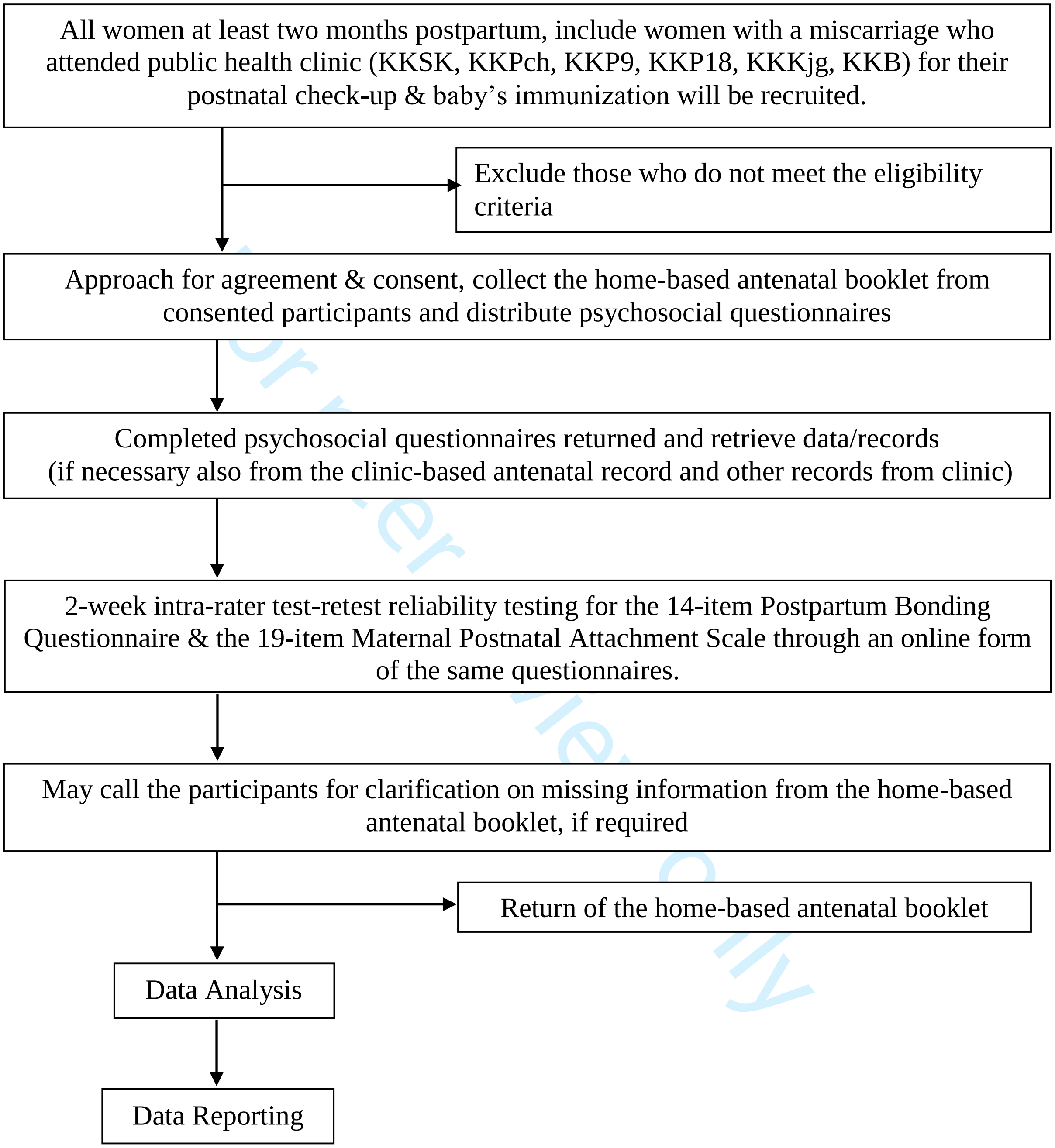

* KKP9= Klinik Kesihatan Precint 9; KKP18= Klinik Kesihatan Precint 18; KKSK= Klinik Kesihatan Seri Kembangan; KKPch= Klinik Kesihatan Puchong; KKKjg= Klinik Kesihatan Kajang; KKB=Klinik Kesihatan Bangi

For peer review only - http://bmjopen.bmj.com/site/about/guidelines.xhtml

STROBE checklist, version2.0, dated 07 February 2022

	Item No	Recommendation	Page No
**Title and abstract**	1	(*a*) Indicate the study's design with a commonly used term in the title or the abstract	3
		(*b*) Provide in the abstract an informative and balanced summary of what was done and what was found	3
**Introduction**			
Background/rationale	2	Explain the scientific background and rationale for the investigation being reported	5-7
Objectives	3	State specific objectives, including any prespecified hypotheses	8
**Methods**			
Study design	4	Present key elements of study design early in the paper	7-8
Setting	5	Describe the setting, locations, and relevant dates, including periods of recruitment, exposure, follow-up, and data collection	8
Participants	6	(*a*) *Cohort study*—Give the eligibility criteria, and the sources and methods of selection of participants. Describe methods of follow-up<br>*Case-control study*—Give the eligibility criteria, and the sources and methods of case ascertainment and control selection. Give the rationale for the choice of cases and controls<br>*Cross-sectional study*—Give the eligibility criteria, and the sources and methods of selection of participants	na
		(*b*) *Cohort study*—For matched studies, give matching criteria and number of exposed and unexposed<br>*Case-control study*—For matched studies, give matching criteria and the number of controls per case	8
Variables	7	Clearly define all outcomes, exposures, predictors, potential confounders, and effect modifiers. Give diagnostic criteria, if applicable	9
Data sources/ measurement	8*	For each variable of interest, give sources of data and details of methods of assessment (measurement). Describe comparability of assessment methods if there is more than one group	8-10
Bias	9	Describe any efforts to address potential sources of bias	na
Study size	10	Explain how the study size was arrived at	11
Quantitative variables	11	Explain how quantitative variables were handled in the analyses. If applicable, describe which groupings were chosen and why	11
Statistical methods	12	(*a*) Describe all statistical methods, including those used to control for confounding	12-13
		(*b*) Describe any methods used to examine subgroups and interactions	12-13
		(*c*) Explain how missing data were addressed	12
		(*d*) *Cohort study*—If applicable, explain how loss to follow-up was addressed<br>*Case-control study*—If applicable, explain how matching of cases and controls was addressed<br>*Cross-sectional study*—If applicable, describe analytical methods taking account of sampling strategy	11-13
		(*e*) Describe any sensitivity analyses	11-13

Continued on next page

For peer review only - http://bmjopen.bmj.com/site/about/guidelines.xhtml

STROBE checklist, version2.0, dated 07 February 2022

**Results**			
Participants	13*	(a) Report numbers of individuals at each stage of study—eg numbers potentially eligible, examined for eligibility, confirmed eligible, included in the study, completing follow-up, and analysed	na
		(b) Give reasons for non-participation at each stage	na
		(c) Consider use of a flow diagram	na
Descriptive data	14*	(a) Give characteristics of study participants (eg demographic, clinical, social) and information on exposures and potential confounders	na
		(b) Indicate number of participants with missing data for each variable of interest	na
		(c) *Cohort study*—Summarise follow-up time (eg, average and total amount)	na
Outcome data	15*	*Cohort study*—Report numbers of outcome events or summary measures over time	na
		Case-control study—Report numbers in each exposure category, or summary measures of exposure	na
		Cross-sectional study—Report numbers of outcome events or summary measures	na
Main results	16	(*a*) Give unadjusted estimates and, if applicable, confounder-adjusted estimates and their precision (eg, 95% confidence interval). Make clear which confounders were adjusted for and why they were included	na
		(*b*) Report category boundaries when continuous variables were categorized	na
		(*c*) If relevant, consider translating estimates of relative risk into absolute risk for a meaningful time period	na
Other analyses	17	Report other analyses done—eg analyses of subgroups and interactions, and sensitivity analyses	na
**Discussion**			
Key results	18	Summarise key results with reference to study objectives	na
Limitations	19	Discuss limitations of the study, taking into account sources of potential bias or imprecision. Discuss both direction and magnitude of any potential bias	14
Interpretation	20	Give a cautious overall interpretation of results considering objectives, limitations, multiplicity of analyses, results from similar studies, and other relevant evidence	na
Generalisability	21	Discuss the generalisability (external validity) of the study results	na
**Other information**			
Funding	22	Give the source of funding and the role of the funders for the present study and, if applicable, for the original study on which the present article is based	3 & 16

*Give information separately for cases and controls in case-control studies and, if applicable, for exposed and unexposed groups in cohort and cross-sectional studies.

Note: An Explanation and Elaboration article discusses each checklist item and gives methodological background and published examples of transparent reporting. The STROBE checklist is best used in conjunction with this article (freely available on the Web sites of PLoS Medicine at http://www.plosmedicine.org/, Annals of Internal Medicine at http://www.annals.org/, and Epidemiology at http://www.epidem.com/). Information on the STROBE Initiative is available at www.strobe-statement.org.

For peer review only - http://bmjopen.bmj.com/site/about/guidelines.xhtml
